# Computational pathology annotation enhances the resolution and interpretation of breast cancer spatial transcriptomics data

**DOI:** 10.1038/s41698-025-01104-3

**Published:** 2025-09-09

**Authors:** Tianyi Li, Qiao Yang, Balazs Acs, Emmanouil G. Sifakis, Hosein Toosi, Camilla Engblom, Kim Thrane, Qirong Lin, Jeff E. Mold, Wenwen Sun, Ceren Boyaci, Sanna Steen, Jonas Frisén, Jens Lagergren, Joakim Lundeberg, Xinsong Chen, Johan Hartman

**Affiliations:** 1 Department of Oncology-Pathology, Karolinska Institutet, Stockholm, Sweden; 2 Department of Clinical Pathology and Cancer Diagnostics, Karolinska University Hospital, Stockholm, Sweden; 3 SciLifeLab, Department of Computation Science and Technology, KTH Royal Institute of Technology, Stockholm, Sweden; 4 Department of Cell and Molecular Biology, Karolinska Institutet, Stockholm, Sweden; 5 SciLifeLab, Division of Immunology and Respiratory Medicine, Department of Medicine Solna, Karolinska Institutet, Center for Molecular Medicine, Karolinska University Hospital, Stockholm, Sweden; 6 SciLifeLab, Department of Gene Technology, KTH Royal Institute of Technology, Stockholm, Sweden

**Keywords:** Computational biology and bioinformatics, Gene expression analysis, Breast cancer, Tumour heterogeneity, Imaging

## Abstract

Breast cancer is a highly heterogeneous disease with diverse outcomes, and intra-tumoral heterogeneity plays a significant role in both diagnosis and treatment. Despite its importance, the spatial distribution of intra-tumoral heterogeneity is not fully elucidated. Spatial transcriptomics has emerged as a promising tool to study the molecular mechanisms behind many diseases. It offers accurate measurements of RNA abundance, providing powerful tools to correlate the morphologies of cellular neighborhoods with localized gene expression patterns. However, the spot-based spatial transcriptomic tools, including the most widely used platform, Visium, do not achieve single-cell resolution readouts, which hinders data interpretability. In this study, we present a computational pathology image analysis pipeline (i.e., computational tissue annotation, CTA) that utilizes machine learning algorithms to accurately map tumor, stroma, and immune compartments within Visium-assayed tumor sections. Using a cohort of 23 breast tumor sections from four patients, we demonstrate that CTA can provide high-resolution annotations on the hematoxylin-and-eosin-stained images alongside the paired sequencing data, support the evaluation of deconvolution methods, deepen insights into intra-tumoral heterogeneity by increasing data analysis resolution, assist with spatially resolved intrinsic subtyping, and enhance the visualization of lymphocyte clones at single-cell resolution. The proposed pipeline provides valuable insights into the complex spatial architecture of breast cancer, contributing to more personalized diagnostics and treatment strategies.

## Introduction

Biological specimens, especially tumor tissues, usually manifest ecosystems with extraordinary cellular complexity and dynamic heterogeneity. Cancer complicacy represents the diversification of the tumor microenvironment (TME)’s cancerous and noncancerous compartments among both spatial and temporal dimensions. This disease complexity, also known as intra-tumoral heterogeneity, is a multifactorial event contributed by but not limited to cancer clonal evolution, various cell types and compositions, their distributions and states within the TME, and cell-to-cell interactions^1^. The active evolution of such biological features is critical in determining disease development, tissue homeostasis, and responses to external stimuli, such as anti-neoplastic therapeutics.

To date, the dominant method for performing such analysis remains bulk sequencing tools, with single-cell sequencing emerging as a strong contender due to its excellent cellular resolution and high-throughput capacity. However, both methods have their limitations. Bulk analysis provides an average of information across cell populations, thus masking cellular heterogeneity. Conversely, a major bottleneck of single-cell sequencing is the lack of contextual information, such as the interactions, coexistence, and communication patterns of individual cells within their native tissue environment.

The advent of spatial transcriptomic (ST) technologies^2,3^ and their continuing development are revolutionizing our understanding of tissue architecture and equipping us with new tools for studying spatial biological events^4^. Current ST approaches are mainly sequencing- or image-based, which have their advantages and trade-offs regarding detection sensitivity, transcriptome-wide profiling capability, spatial resolution, capturing area size, and compatibility with various types of materials^4^. Among them, the 10X Visium technology is a popular commercially available choice, well known for its product maturity, and has thus far generated the most spatial gene expression (GEX) datasets^4^. Considering the relatively large spot size of the Visium assay (55μm in diameter), data from individual spots generally represent aggregates of multiple cells of distinct types, yielding mixed gene expression profiles, limiting efforts to understand how individual cells contribute to the spatial microenvironment. The growing demand for higher spatial resolutions has stimulated the development of matching data analytical tools. Despite the availability of imaging as a modality to identify single-cell level details in parallel with transcriptomic tools, most of the methodological advances have focused only on the GEX data in efforts to model single-cell relationships or predict other omics data in ST analysis^5–7^. The massive amount of morphological data in the histopathology images is often overlooked but could be used to enhance the actual readout of the GEX data.

For standard formalin-fixed paraffin-embedded (FFPE) sections, morphology-assisted methods are already emerging for synergistic analysis of Hematoxylin and Eosin (H&E) images and spatial data^8^. In contrast, the fresh frozen-based ST assay poses more technical challenges for high-quality image analysis. For instance, it often uses thicker tissue sections (10μm) and has ambiguous morphology due to the lack of dehydration and fixation steps prior to staining. However, fresh-frozen tissues typically preserve better RNA quality and allow the generation of raw sequences by Visium, which is critical for the development of new spatial technologies, such as the recently reported Spatial VDJ^9^ and Spatial ATAC^10^.

In this study, we established a pipeline to annotate Visium-matched H&E-stained images from frozen sections at single-cell resolution for breast tumors. Subsequently, we demonstrated how to use it to enhance spatial transcriptomics data interpretation when analyzing intra-tumoral heterogeneity. The method primarily employs a machine learning based object classifier incorporated into the QuPath platform^11^, enabling us to precisely infer the major cell types within the TME for individual cells from the H&E images. Using our pipeline, we further matched their positional information back to the corresponding Visium spot IDs. By analyzing the breast cancer ST data generated in-house alongside paired H&E images in detail, we showed that our computational tissue annotation (CTA) was capable of supporting the performance evaluation of various deconvolution methods for our dataset, enhancing the manifestation of GEX clustering analysis, assisting in cancer copy number variation (CNV) calling, studying the spatial intrinsic subtypes of breast cancer, and improving the visualization resolution of paired immune cell clones^9^ within the tissue context.

## Results

### Study overview

**Fig. 1:**
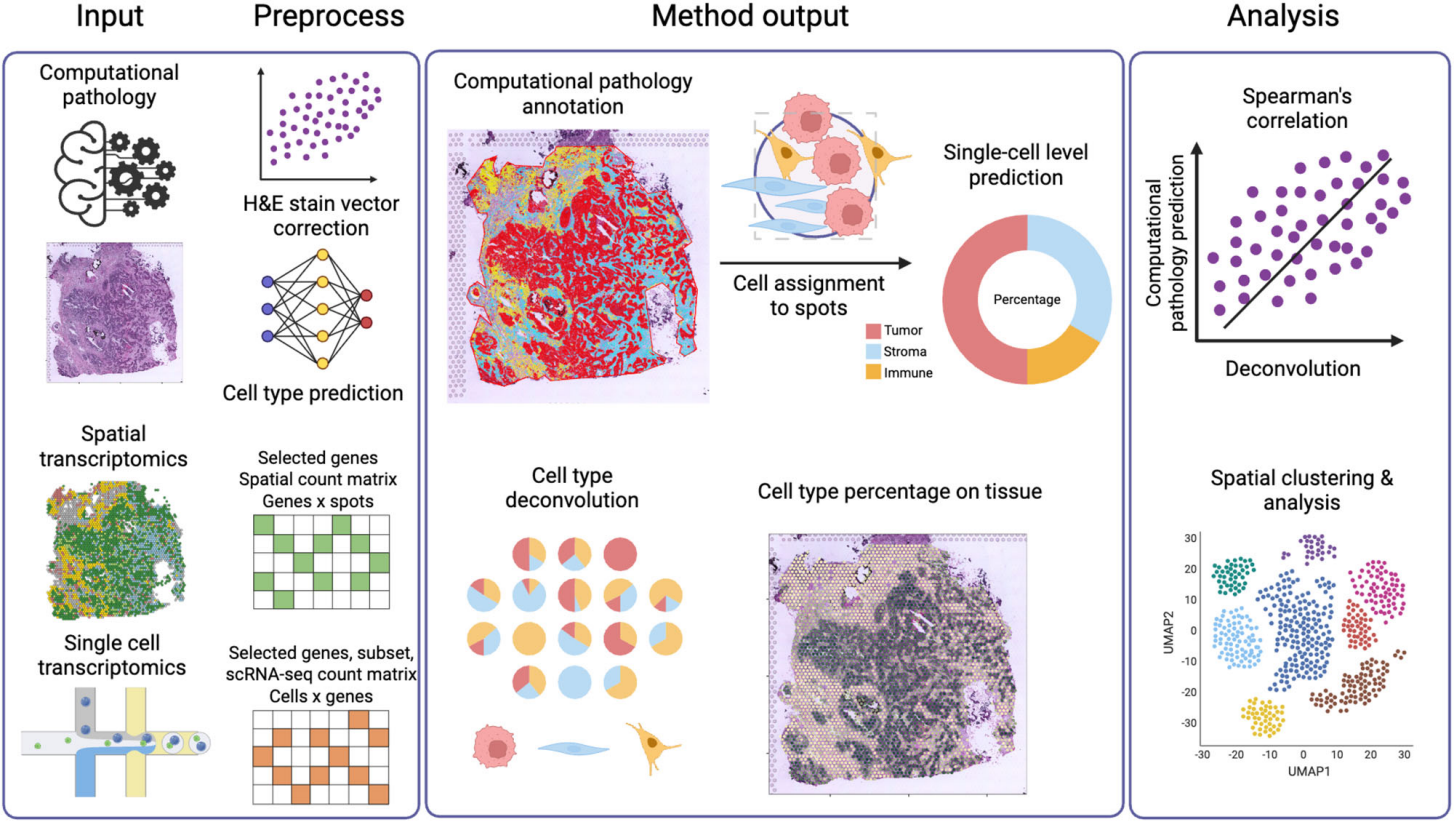
Study overview. Experimental setup of joint analysis of breast cancer sections using spatial transcriptomics and our computational pathology approach. Computational tissue annotation was performed on matched H&E images from Visium spatial transcriptomics analysis supported by the QuPath (version 0.5.1) platform. After stain vector correction, cell types were annotated by an object classifier using the random trees algorithm. In parallel, data from single-cell transcriptomics were downloaded and used as a reference to deconvolute spatial transcriptomics data through seven different methods. Spearman’s correlation coefficient was used to measure the performance of deconvolution methods. Subsequently, gene expression clustering and other downstream analyses were carried out using the outperformed deconvolution method, assisted by the computational tissue annotation in different ways. Created in BioRender. Li, T. (2025) https://BioRender.com/n2mhmke.

In this study, we proposed a user-friendly analysis pipeline and showcased its application in enhancing the resolution of traditional spatial transcriptomics data analysis. We established a standard operating procedure for performing CTA on the H&E-stained breast tumor frozen sections paired with the Visium data (Supplementary Fig. 1). In brief, after H&E stain vector correction, we segmented the nucleus, followed by training and applying an object classifier using a random trees algorithm that predicts cell type based on tissue morphology and histological features, which is powered by the QuPath platform^11^. The features used for training the object classifier were provided in Supplementary Data 1. A board-certified pathologist also reviewed and evaluated the annotations to ensure the reliability of the data. Subsequently, we aligned the CTA annotations to the Visium spots and quantified the count of each cell type. In parallel, the spatial transcriptomics data were deconvoluted using a publicly available reference single-cell RNA sequencing (scRNA-seq) dataset^12^ with different methods, and their performances were assessed using Spearman’s correlation analysis with the results from CTA. Lastly, we presented how to integrate our proposed CTA pipeline into routine spatial transcriptomics analysis for improved comprehension of breast cancer intra-tumoral heterogeneity (Fig. 1).

### Computational pathology approach provides high-resolution tissue annotations on H&E images

**Fig. 2:**
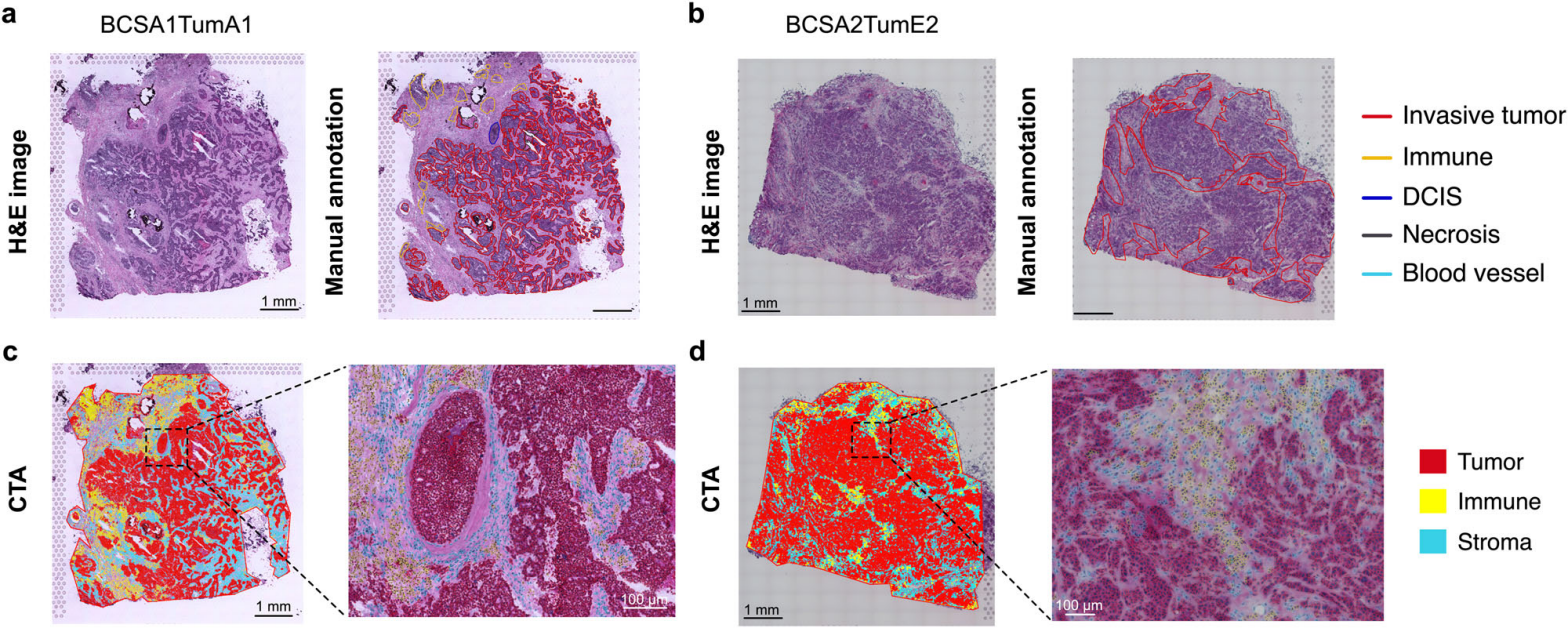
Computational pathology provides high-resolution annotations on H&E images. H&E image from BCSA1TumA1 (**a**) and BCSA2TumE2 (**b**) with their paired manual annotation by pathologists. The red label outlines invasive tumors; yellow marks immune areas; dark blue indicates DCIS; black draws necrosis areas; and sky blue suggests blood vessels. Scale bar 1 mm. Computational tissue annotations for BCSA1TumA1 (**c**) and BCSA2TumE2 (**d**) using the object classifier trained with a random trees algorithm on the whole (left) and higher magnification image (right). Red indicates tumor; yellow highlights immune; and blue illustrates stroma. A and E represent the sample ID denoting the sampling area from each patient and the numbers 1 and 2 represent the number of technical replicates on the tumor regions A and E, respectively.

H&E images (*n* = 23) with paired spatial transcriptomics data (Supplementary Fig. 1) were annotated manually by board-certified pathologists and computationally by CTA. In brief, the CTA approach allowed the prediction of individual tumor, immune, and stroma cell identities from the H&E images. Results from two breast cancer samples were shown as examples, where a pathologist provided detailed information on the invasive tumor (red), immune (yellow), ductal carcinoma in situ (DCIS, dark blue), necrosis (black), and blood vessel (sky blue) on the images (Fig. 2a, b). However, the tumor regions were often infiltrated with other cell types, and the stroma areas were very challenging to annotate manually in detail. In the CTA-annotated images, tumor cells were marked in red, immune cells in yellow, and stroma cells in blue (Fig. 2c, d). Our results showed that the classifications were highly consistent with the pathologist’s annotations and also at single-cell resolution.

To further investigate the accuracy of our CTA pipeline, we obtained a HER2-positive breast cancer sample assayed with the Xenium platform from 10X Genomics and analyzed its paired H&E image^3^. Surveying the Xenium probe-based in situ sequencing and imaging data, we found high agreement between three cell type markers (*ERBB2*-tumor, *PTPRC*-immune, and *PDGFRA*-stroma) and our CTA outcomes, suggesting its robustness for detecting FFPE sections as well (Supplementary Fig. 2). Overall, our computational pathology approach can provide precise and high-resolution annotations on the H&E images from both Visium and Xenium platforms.

### Computational tissue annotation assists the performance evaluation of deconvolution methods

**Fig. 3:**
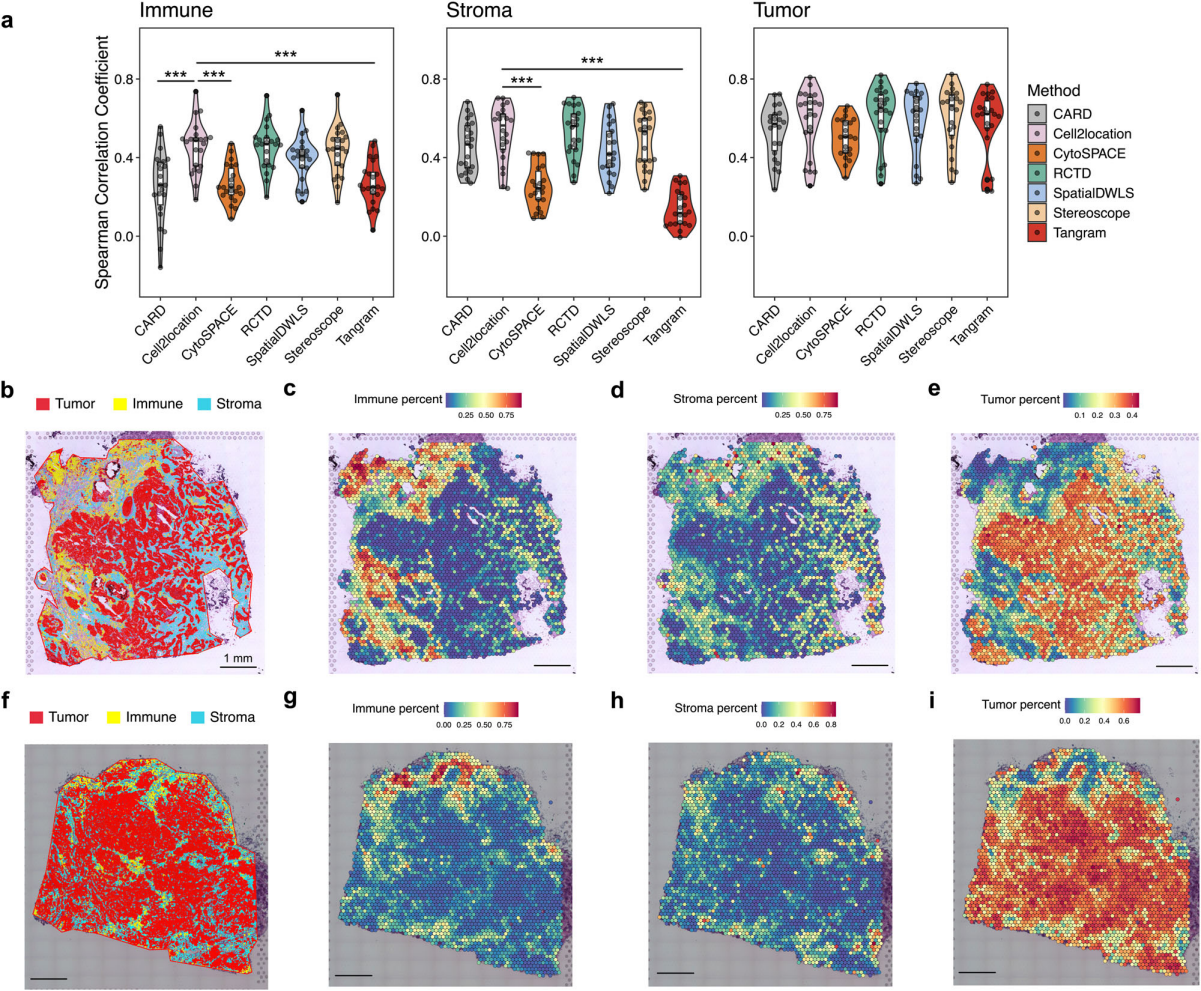
Assess the performance of deconvolution methods by referencing computational tissue annotation outcomes. **a** Spearman’s correlation coefficient of comparison between results from single-cell level classification by CTA and different deconvolution methods (22 Visium-assayed tumor regions from 4 patients). One sample (BCSA3TumB2) was excluded from CTA analysis due to a mismatch of image metadata. BCSA4TumA1 was excluded from SpatialDWLS due to a filtering issue in deconvolution. Statistics were performed using the Kruskal–Wallis with Dunn’s post hoc test and Benjamini–Hochberg’s correction in RStudio. The violin plots outline the distribution, and the boxplots show the 25^th^ and 75^th^ percentiles, with the middle lines representing the median values. Asterisks (*) outline the statistical significance between each method and Cell2location. ***p < 0.001. Cell-level annotation by CTA on BCSA1TumA1 (**b**) and BCSA2TumE2 (**f**) sections. Red indicates tumor; yellow, immune; and blue, stroma. Scale bar 1 mm. The percentage of immune (**c**, **g**), stroma (**d**, **h**), and tumor (**e**, **i**) cells predicted by Cell2location deconvolution for both sections is also shown. The color indicates percentage of the corresponding cell type, whereas blue represents a low percentage and red a high percentage. Scale bar 1 mm.

Sequencing-based spatial transcriptomics platforms offer valuable full-transcriptome data, but many still lack single-cell resolution, creating a strong demand for methods to decompose cellular composition. Consequently, cellular deconvolution techniques using scRNA-seq as a reference have advanced rapidly. Despite earlier efforts to benchmark existing spatial transcriptomics-compatible deconvolution methods using both simulated and real-world datasets^13,14^, selecting the most appropriate deconvolution method to analyze complex diseases such as cancer remains challenging. Most of the available deconvolution methods rely heavily on the expression of a limited panel of cell-type markers to decompose cell-type composition. In contrast, our image-based CTA provides primary cell identity information by directly profiling cellular histological morphology, offering a complementary angle to understand the tissue composition in combination with the molecular data. In this study, to identify and apply the most suitable deconvolution method among CARD^15^, Cell2location^16^, RCTD^17^, Stereoscope^18^, CytoSPACE^19^, SpatialDWLS^20^, and Tangram^21^, we used the image-based CTA result as a reference to assess their performance. By scaling and aligning each Visium spot position onto the annotated image, we generated an output of cell composition percentages (tumor, immune, and stroma) using the CTA annotations that was similar to the traditional deconvolution readout. Notably, CTA was developed also to generate cell counts, which could then be used to refine deconvolution by defining the number of cells expected in each spot. A publicly available scRNA-seq dataset from breast tumors was used to deconvolute our Visium data for the selected methods^12^. We aggregated the deconvolution results of 29 cell types defined by the dataset to tumor, immune, and stroma levels, further correlated them with CTA results, and calculated Spearman’s correlation coefficients. As shown, we observed moderate to strong correlations between most of the deconvolution methods and our CTA on tumor cells, with Cell2location, RCTD, and Stereoscope standing out with their median coefficient >0.65 (Fig. 3a). These outperforming methods were also prominent for the stroma and immune cells (median coefficient between 0.53–0.56 and 0.45–0.49, respectively). CytoSPACE and Tangram revealed significantly lower performances (*p* < 0.0001) compared to Cell2location in both categories, while CARD also lagged for immune cell classification (*p* < 0.0001) (Fig. 3a). In summary, Cell2location, RCTD, and Stereoscope showed the highest concordance between deconvolution and CTA results for our dataset among all tested methods. We picked Cell2location for the downstream analysis. The plotted deconvolution results obtained from Cell2location showed high agreement with the CTA on the same breast cancer sections (Fig. 3b–i), which was further supported by the spatial expression of cell type markers (i.e., *PTPRC* for immune, *FAP* for stroma, and *EPCAM* for tumor) in the same tumor regions (Supplementary Fig. 3).

### Computational tissue annotation enhances the resolution of spatial transcriptomics analysis

**Fig. 4:**
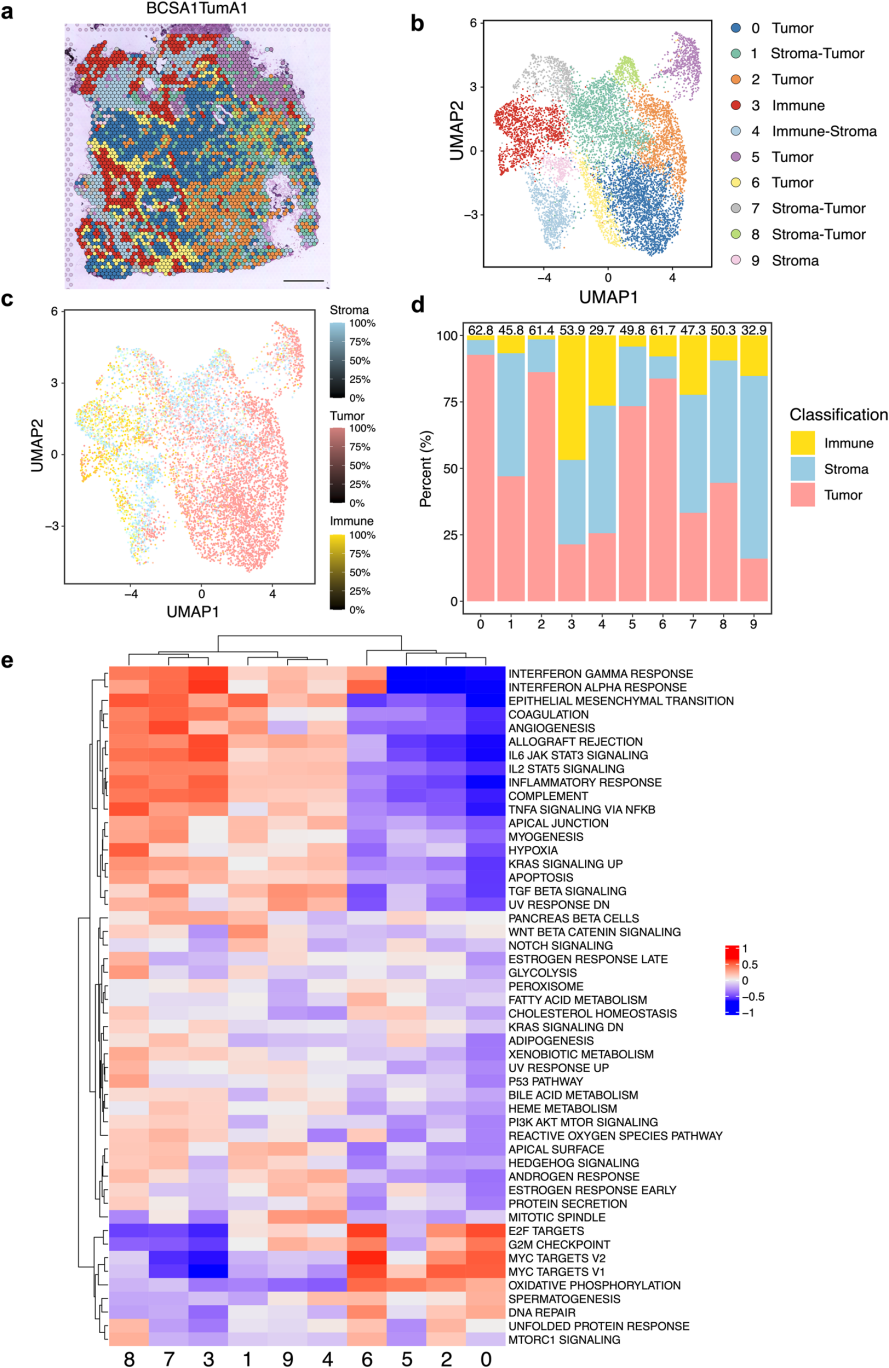
Analysis of TNBC samples identifies transcriptional intra-tumoral heterogeneity patterns. **a** Spatial GEX clusters are shown on the BCSA1TumA1 section after the rPCA integration of samples from both regions A and B. Scale bar 1 mm. **b** Uniform manifold approximation and projection (UMAP) of identified GEX clusters after integrating data from all 4 sections from regions A and B. The color codes represent different clusters and are shown as a sidebar. **c** UMAP of the BCSA1 sample colored by the percentage of stroma, tumor, and immune cells classified by CTA. **d** Percentage of immune, stroma, and tumor predicted by CTA for each GEX cluster within BCSA1 samples. Immune cells are shown in yellow, stroma cells in blue, and tumor cells in pink. The spot density, calculated by the total number of cells/nuclei annotated by CTA divided by the number of spots in each cluster, was displayed on top of each stacked bar (cells/spot). **e** Gene set variation analysis (GSVA) on aggregated, cpm-normalized, and log-transformed count matrix in each cluster against the hallmark gene sets. The color scale indicates pathway activity predicted by GSVA, where blue represents low and red indicates high activity.

In this section, we demonstrated how our proposed pipeline could improve data analysis by providing quantitative insights into tissue composition, and how it could be integrated smoothly into traditional Visium analysis. Each tumor included in the study was sampled multiple times (between two to five separate biological specimens), permitting us to investigate the spatially resolved intra-tumoral heterogeneity within these patients (Supplementary Fig. 1). The triple-negative breast cancer (TNBC) tumor BCSA1 was divided into regions A and B upon OCT-embedding and used for further Visium experiments. After integrating the sequencing data from these two regions, including replicates from adjacent sections, we identified 10 unique GEX-based clusters (Fig. 4a, b, Supplementary Figs. 4, 5). To determine the cluster identity with ambiguous expression of cell-type markers, we retrieved results from CTA and plotted the distribution and composition of stroma, immune, and tumor from each Visium spot, matching with the uniform manifold approximation and projection (UMAP) for specific GEX clusters (Fig. 4b–d). Among the 4 GEX-based tumor clusters (clusters 0, 2, 5, 6), CTA confirmed that their tumor fraction made up at least 70% of the total cells. Likewise, 47% of immune (cluster 3) and 69% of stroma (cluster 9) made up their own GEX clusters. The clusters with mixed GEX cell type profiles (clusters 1, 4, 7, 8) were also identified by the CTA approach, with none of the individual cell types occupying more than half of the total populations (Fig. 4c, d). Similar findings between CTA and GEX clustering analysis were observed for the HER2-positive BCSA2 and BCSA3 tumors, where a gradient of different cell type mixtures constituted the individual GEX-defined clusters identified following the same analysis procedures (Supplementary Figs. 6a–d, 7, 8, 9a–d, 10, 11). Notably, CTA could help recover crucial cell identity information that sometimes might be missed by traditional analysis methods based on top-expressed genes, which might result in a biased annotation of gene clusters. Taking BCSA3 cluster 2 as an example, although various immunoglobulin-encoding genes, which expressed by B lineage cells, topped the expression ladder, CTA uncovered a substantial fraction of stromal populations within the cluster (Supplementary Figs. 9, 10). Similarly, the BCSA2 cluster 5 revealed a mixed TME composition, with tumor, immune, and stromal populations contributing almost equally according to CTA classification (Supplementary Fig. 6). Immunoglobulin-encoding genes were again found to be the most abundantly expressed (Supplementary Fig. 7).

Subsequently, we performed gene set variation analysis (GSVA) on the aggregated and normalized count matrix using the pseudo-bulk method against the Hallmark gene set^22,23^ to validate cell type annotation and to study the biological differences among the identified clusters. For BCSA1, GSVA demonstrated a similar profile between tumor clusters 0, 2, and 6, where high activity in pathways related to proliferation, cell cycle, oxidative phosphorylation, and MYC-targets was observed (Fig. 4e). Uniquely, tumor cluster 5 presented a pattern of dampened proliferation and DNA repair characteristics but moderately upregulated metabolism signaling such as adipogenesis and cholesterol homeostasis (Fig. 4e). Worth mentioning, this cluster localized within both the identified DCIS and invasive cancer areas in TumA region that were well separated from other tumor clusters (Figs. 2a, 4a, Supplementary Fig. 5a). For the HER2-positive BCSA2 and BCSA3, cancer cell-enriched clusters for both tumors (BCSA2-clusters 0, 1, 9 & BCSA3-clusters 3, 4, 7) showed close relationships and resemblance (Supplementary Figs. 6e, 9e). The non-cancer clusters in all the samples, including those identified as tumor-mixed, were clearly segregated by GSVA from the tumor-enriched clusters. They usually displayed blended biological signatures, matching their more heterogeneous TME compositions (Fig. 4d, e, Supplementary Figs. 6d, e, 9d, e). Furthermore, we also identified some GEX clusters with spatial distribution preferences. While the localized gene expression patterns were relatively homogenous among BCSA1 regions, a greater level of heterogeneous allocation of GEX clusters was found in BCSA2&3 (Supplementary Figs. 5, 8, 11).

**Fig. 5:**
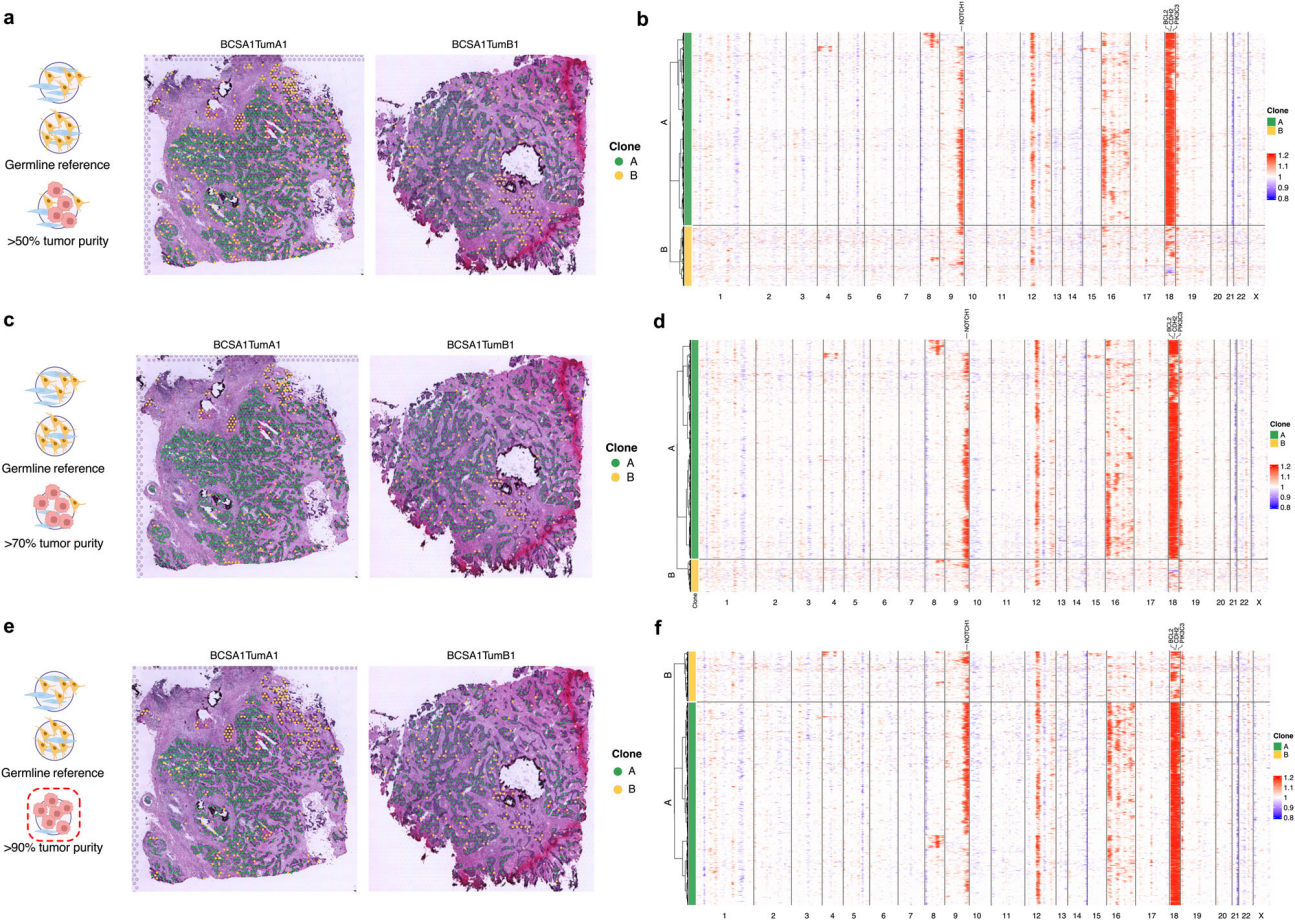
Spatial determination of CNV status to identify clonal events in the TNBC tumor. Spatial visualization of tumor clones on the BCSA1 TumA1 and TumB1 sections using spots with at least 50% (**a**), 70% (**c**), and 90% (**e**) of tumor purity defined by CTA. Genome-wide CNV analysis for each spot with at least 50% (**b**), 70% (**d**), and 90% (**f**) of tumor cell fractions. The color indicates scaled expression levels, with blue representing copy number loss and red indicating copy number gain. Clonal grouping of spots was defined by hierarchical clustering. The chromosome numbers are displayed at the bottom of the heatmap.

Lastly, we studied the copy number variations (CNVs) across tumor regions using the spatial inferCNV approach^5^ and showcased the usage of CTA to refine our copy number predictions. Based on our CTA classifications, we selected all the spots without tumor cell count and used them as the germline reference for CNV estimation. Subsequently, we identified spots composed of at least 50%, 70%, and 90% of tumor cells according to CTA outputs and performed the copy number analysis. To minimize the potential selection bias, hierarchical clustering was performed on the scaled copy number profile to determine the number of distinct clones and their spatial distribution was visualized on the H&E images. In BCSA1 samples, the CNV events were primarily identified on chromosomes (chr) 9, 12, 16, and 18, from where we further classified them into two major clones with the hierarchical clustering using all three different tumor purity cutoff values (Fig. 5). With the increase of the tumor purity, the spots with no CNV events were filtered out, and the results revealed a dominant chr18 and a subtle chr16 amplification in clone A (Fig. 5f), which aligned with the GEX analysis, and were mainly composed of spots in the tumor-rich clusters 0, 2, and 6. In contrast, DCIS and its adjacent invasive areas (cluster 5) primarily belonged to clone B, which lacked amplifications on chr16 and also had the amplifications on chr18 largely absent (Figs. 2a, 4, 5, Supplementary Fig. 14). In samples from a HER2-positive tumor (BCSA2), we could only identify one major clone distributed throughout all the tumor areas under all the cutoff settings (Supplementary Fig. 12). On the other hand, the CNV prediction was substantially improved with the increase of tumor purity threshold in BCSA3 samples (Supplementary Fig. 13). Under the cutoff of 50%, four major clones were defined in BCSA3 samples with notable CNV events shown on chr 1, 7, 8, 12, 13, 16, and 17 (Supplementary Fig. 13b). However, only a limited number of events were displayed in clone D, and we observed a relatively diffused spatial distribution of it compared to the other three clones (Supplementary Fig. 13a, b). With stricter inclusion criteria defined by CTA, this clone disappeared. The analysis showed a cleaner separation in their spatial distribution, and the heatmap exhibited a clear boundary among the three clones inferred using 70% and 90% cutoff (Supplementary Fig. 13c–f). Interestingly, tumor clones in BCSA3 were seemingly distributed with regional preferences. TumB1 and TumC1 were mostly occupied by clone B, while clone A dominated TumD1. An evident boundary could be observed in the section dividing clone A areas from clone C, which was seen exclusively in TumA1 (Supplementary Figs. 13e). Interestingly, while clone A was composed evenly of the GEX-based tumor clusters (3, 4, 7) and the mixed cluster 1, clone C was almost exclusively made up of tumor cells from cluster 3 (Supplementary Fig. 14). In summary, incorporating CTA annotation as a quantitative standard for spatial inferCNV analysis can help predict copy number events and define clones, leading to a deeper understanding of breast cancer complexity.

### Spatially resolved breast cancer intrinsic subtyping supported by computational tissue annotation

**Fig. 6:**
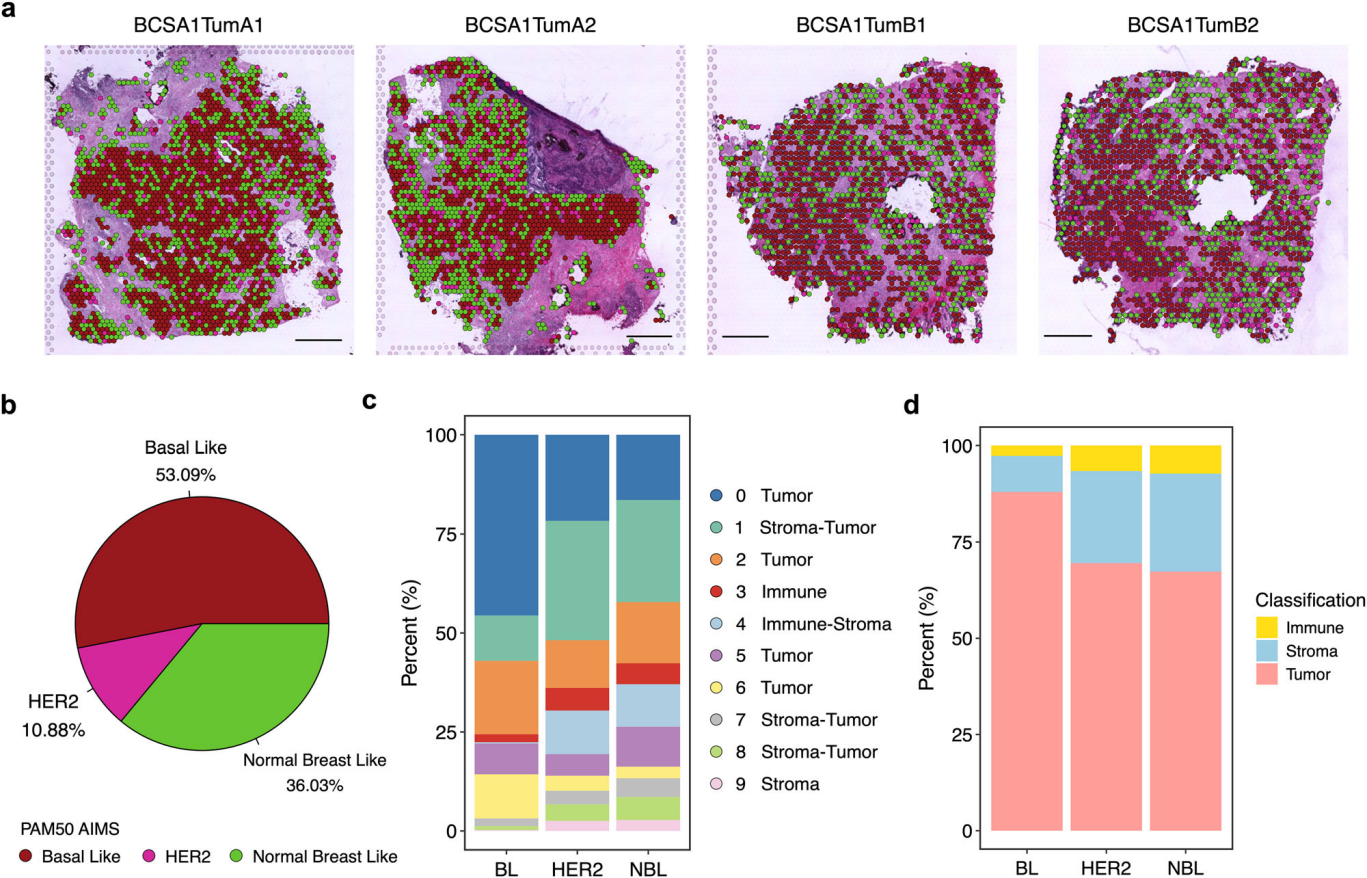
Heterogeneity of spatially resolved breast cancer intrinsic subtypes in the TNBC tumor. **a** Spatially resolved intrinsic subtypes predicted by the AIMS method across all four sections of BCSA1. The folded area in TumA2 was excluded from the analysis. The scarlet color code indicates Basal-Like (BL), fuchsia pink represents HER2, and green denotes Normal Breast-Like (NBL). Scale bar 1 mm. **b** Pie chart showing the overall distribution of predicted intrinsic subtypes from all BCSA1 sections. **c** Stacked columns displaying the composition of GEX clusters within the BL, HER2, and NBL subtypes. **d** Percentage of immune, stroma, and tumor cells in each intrinsic subtype predicted by CTA. Immune in yellow, stroma in blue, and tumor in pink.

Breast cancer can be classified into five intrinsic molecular subtypes with prognostic and therapeutic implications using the PAM50 gene signature: luminal A (LumA), luminal B (LumB), HER2-enriched (HER2), basal-like (BL), and normal-breast-like (NBL)^24–27^. We predicted the intrinsic molecular subtypes using the Absolute Intrinsic Molecular Subtyping (AIMS) method on our spatial transcriptomic data^28^. The Cell2location algorithm was applied to deconvolute the data, excluding spots with less than 20% of epithelial cells to ensure that intrinsic subtyping was performed only for tumor-containing areas. We further filtered the remaining spots based on our CTA classification, excluding those without identified tumor cells. For the TNBC BCSA1, we eventually identified a heterogeneous pattern of intrinsic subtypes distributed within each region and across sections, with 53.09% BL, 36.03% NBL, and, interestingly, 10.88% HER2 (Fig. 6a, b). To determine if any GEX-based clusters contributed specifically to certain intrinsic subtypes, we also measured the composition of the 10 clusters in each subtype. Notably, BL spots were largely occupied by tumor cell-rich clusters (0, 2, 5, 6). Higher fractions of immune, stroma, and mixed clusters (1, 3, 4, 7, 8, 9) were observed within the HER2 and NBL subtypes. This was further supported by our CTA analysis, which showed HER2 and NBL spots containing higher percentages of immune and stroma cells compared to the BL subtypes (Fig. 6c, d).

For the HER2-positive BCSA2 and BCSA3, most tumor areas were identified as HER2-enriched, with 87.35% and 58.59%, respectively. Around 10% NBL and less than 2% LumA constituted the rest of BCSA2 spatially, while BCSA3 showed a broader spectrum of intrinsic subtype distributions with approximately 29.04% NBL, 11.39% LumA, and 0.97% LumB (Supplementary Figs. 15a, b, 16a, b). The tumor GEX clusters (0, 1, 9) overlapped with at least 75% of the HER2 and LumA spots in BCSA2, while the stroma and immune fractions contributed more to the NBL subtype (Supplementary Fig. 15c, d). However, BCSA3 showed a divergent pattern in the composition of identified subtypes, with the tumor clusters (3, 4, 7) occupying the LumB spots almost exclusively and gradually decreasing in the LumA, HER2, and NBL areas (Supplementary Fig. 16c, d). Again, such findings were consistent with our CTA analysis using the H&E images alone (Supplementary Figs. 15c, d, 16c, d).

### Computational tissue annotation enhances the visualization resolution of spatially identified lymphocyte clones

**Fig. 7:**
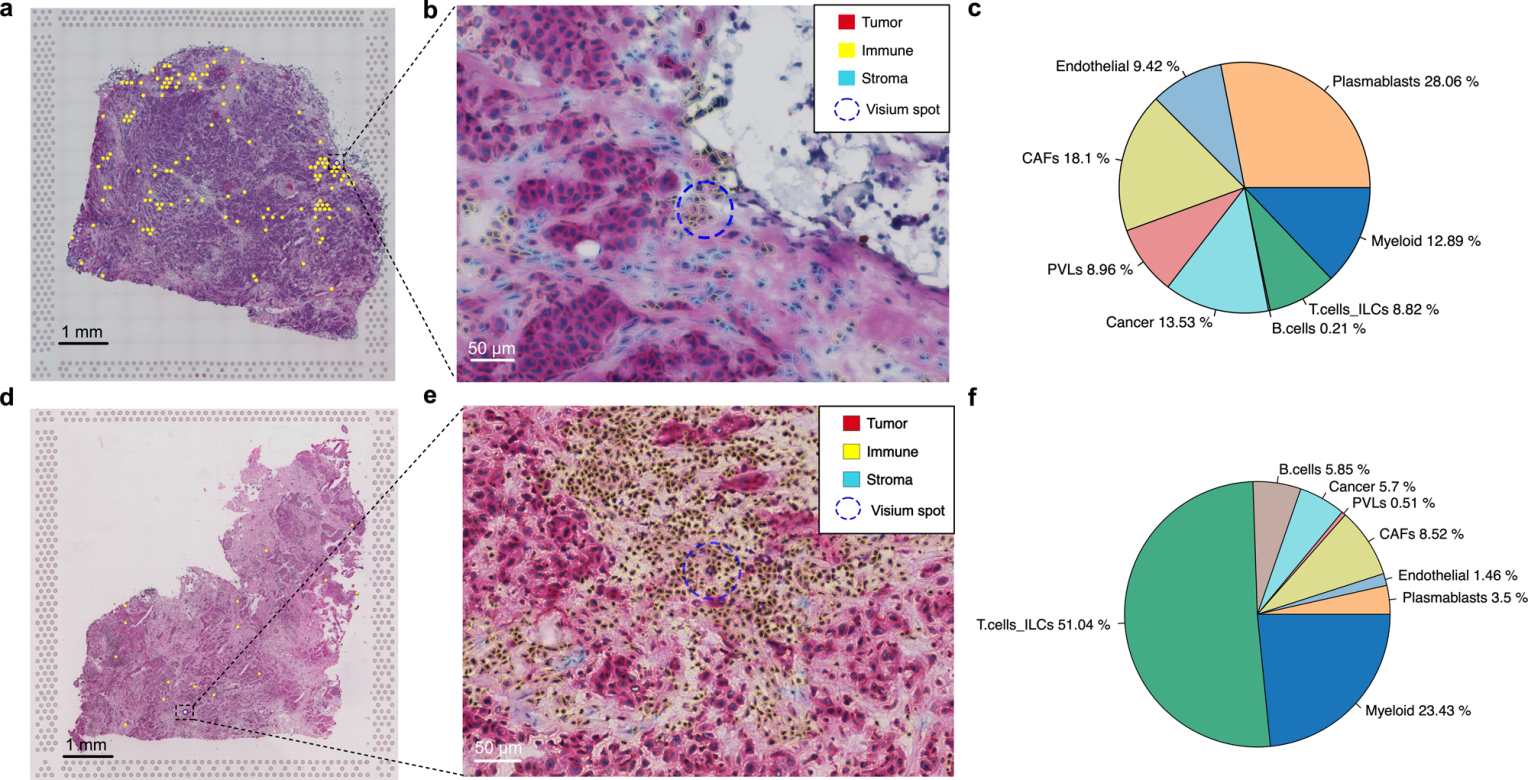
Computational tissue annotation enhances the visualization resolution of spatially identified lymphocyte clones. **a** Spatial distribution of BCSA2TumE2 B-cell clone 0 indicated by the yellow dots representing individual Visium spots. Scale bar 1 mm. **b** Magnified H&E image showing a selected spot enriched with B-cell clone 0. The blue dotted-line circle marked the position of the selected Visium spot, while colors around the nucleus represent the annotation of the cells. Red indicates tumor cells, yellow refers to immune cells, and blue implies stroma cells. Scale bar 50 μm. **c** Deconvolution outputs of the exact spot showing in (**b**) with B-cell clone 0 using the Cell2location method. **d** Spatial distribution of BCSA3TumA1 T-cell clone 61 indicated by the yellow dots representing individual Visium spots. Scale bar 1 mm. **e** Magnified H&E image showing a selected spot enriched with T-cell clone 61. The blue dotted-line circle marked the position of the selected Visium spot, while colors around the nucleus represent the annotation of the cells. Red indicates tumor cells, yellow refers to immune cells, and blue implies stroma cells. Scale bar 50 μm. **f** Deconvolution outputs of the exact spot showing in (**e**) with T-cell clone 61 using the Cell2location method. CAFs cancer-associated fibroblasts, PVLs perivascular-like cells, ILCs innate lymphoid cells.

Taking advantage of the existing B and T lymphocyte clonal information of BCSA2 and BCSA3 samples^29,30^, we were able to further unravel the tumor-immune interactions within the TME at an even higher spatial resolution when combined with CTA analysis. Examples of specific B and T cell clones were plotted on the CTA annotated H&E images of BCSA2TumE2 (Fig. 7a, b) and BCSA3TumA1 (Fig. 7d, e) regions. Notably, both the B cell clone and the T cell clone were confirmed to reside within immune cell-rich spots from the magnified annotated images, which were either positioned on the tumor border or within a direct contact range to tumor cells (Fig. 7a, b, d, e). Moreover, in accordance with our image-based assessment, the cell type composition predicted by deconvolution analysis also pointed out that both spots were occupied dominantly by immune cell populations led by plasmablasts and T cells/innate lymphoid cell populations, respectively (Fig. 7c, f). The full list of spots detected with the selected B and T cell clones and their compositions predicted by deconvolution and CTA can be found in Supplementary Data 2.

## Discussion

Microscopic examinations of histological sections and protein-based biomarkers have been the cornerstone of cancer diagnostics and treatment decisions. However, these tools are insufficient for assessing the heterogeneous nature of cancer diseases, which plays a decisive role in modern cancer therapy design^31^. Computational pathology is an emerging field that facilitates clinical decisions by comprehensively interpreting complex pathological images using machine learning algorithms, allowing novel opportunities to improve cancer care^32–36^. With the discovery and growing popularity of spatial profiling technologies^37^, the utility of established histological techniques has largely been overshadowed by transcriptomic data, despite the potential to combine both approaches as a single tool. Here, we proposed a user-friendly computational pathology pipeline (CTA) that provides single-cell level annotations based on machine learning algorithms powered by the QuPath platform on the H&E images paired with the ST data. We presented how our pipeline could be integrated into the current spatial transcriptomics data analysis for better elucidation of complex tumor biology within tissue context at an improved resolution.

Unlike the diagnostic H&E slides that are usually stained and scanned with automated instruments, images from spatial assays are often subjected to manual protocols with individual sample processing adaptations and microscopy preferences during image acquisition. These technical factors pose challenges for nucleus segmentation, for example, hampering the performance of the watershed algorithm used by QuPath^11^, which leads to less precise predictions of the nucleus boundary and cell counts. However, these effects could be mitigated by fine-tuning the cell detection parameters used in the algorithm and/or by applying alternative algorithms such as a convolutional neural network^38^ to improve segmentation. In addition, as with many machine learning-based image analysis approaches, our CTA pipeline requires manual annotations for model training^39^, which may introduce human bias into the prediction outcomes. To help mitigate this potential bias, the random tree algorithm incorporates multiple histological features (*e.g*., nuclear area, cell area, circularity, hematoxylin and eosin staining optical density) to construct an object classifier for cell type classification. Despite such limitations, our CTA presented a robust procedure in recognition and separation of cancer and other TME components at single-cell resolution from the H&E images of OCT-embedded frozen breast tumors (Fig. 2). Moreover, our CTA results showed high degrees of agreement with the manual annotations by pathologists from the same sections, and from results of a breast cancer sample sequenced using the image-based Xenium ST platform, which provides transcript positional information at the sub-cellular level (Supplementary Fig. 2).

Although many spatial deconvolution methods are available in the field^13,14,40^, and a handful of benchmark studies have been carried out to evaluate their performance, it is still difficult for researchers to decide the most fitting method for individual projects and datasets focusing on distinct diseases or biological events. In this study, we proposed using our CTA results as an extra evaluation parameter to find the deconvolution method with the highest correlation with tissue morphology for a given dataset, in our case, breast cancer sections with Visium data. In general, the CTA outputs showed good concordance with the deconvolution results and the expressions of cell-type markers. Using it as a reference, we identified three outperformed methods (Cell2location, RCTD, and Stereoscope) among the seven commonly applied tools tested in our cohort (Fig. 3). Generally, deconvolution methods rely on highly expressed genes or differentially expressed genes to annotate cell types. As a consequence, the choice of marker panel is crucial to method performance^41^. Interestingly, our results revealed a comparable performance between tool that require an input of a predefined marker list (i.e., Stereoscope) and those that use their built-in selection strategies (i.e., Cell2location and RCTD) when comparing the results to those obtained from CTA. We chose Cell2location for downstream analyses due to its slightly higher median correlation coefficients in immune and stroma fractions (Fig. 3).

While most samples exhibited moderate to strong correlations between deconvolution results and CTA output, those characterized by extensive fibrotic regions generally exhibited diminished concordance. Tumor cells are known to display pronounced eosin staining in the H&E images. Consequently, collagen-rich areas, which are also intensely stained by eosin, may introduce noise that compromises tumor fractions prediction. Furthermore, we hypothesized that suboptimal tissue quality might adversely affect sequencing quality, leading to the exclusion of many spots during quality control. This reduction in usable data points diminished the robustness of correlation analysis, resulting in weaker correlations.

Worth noticing, many deconvolution methods use the embedded algorithm to estimate cell counts from RNA abundance, with a few tools provide options for the users to input the expected number of cells or nuclei in each spot^15–21^. Since the high transcriptional activities and RNA abundance of tumor cells usually lead to an overestimation of cell counts compared with non-cancerous TME populations, utilizing the image-based quantification by CTA could potentially improve the deconvolution performances.

Not surprisingly, even the most tumor-enriched GEX clusters had a gradient of mixed non-tumorous components that would be neglected if only looking at their top-expressed transcripts (Fig. 4, Supplementary Figs. 6 and 9), which is the technical constraint of spot-based ST methods such as Visium. Until recently, most studies applying spatial techniques have utilized spot-level tissue annotations or manually drawn the area with distinct histological features. While manual annotation could sometimes provide more tissue structural information on the images (i.e., areas of DCIS, necrosis, and blood vessels), performing a detailed and precise manual annotation is time-consuming and laborious. In addition, such management does not reach single-cell resolution, making it challenging to incorporate the readout into ST data analysis. In this study, we presented the use of our CTA output from the individual spot to help with the clustering annotations and tumor clone predictions with spatial inferCNV. More specifically, we first selected the germline reference by excluding spots with any tumor count identified by CTA. In such management, we could minimize the probability of cancer cell contamination. Subsequently, the spots included in the CNV analysis were selected based on tumor purity quantified by CTA. Our results showed that increasing the tumor purity threshold could significantly refine the performance of CNV assessment, leading to a more confident calling and a clearer pattern of copy number events. Notably, the detected copy number observations displayed a good concordance with that of the whole-exon sequencing (WES) data obtained from the same samples^42^. Apart from that, we identified an interesting clone-to-expression pattern in BCSA1 tumors that involved both DCIS and invasive areas, which distinguished the specific GEX cluster 5 from the other cancer clusters by both the CNV event on chr 16 and 18 and the pathway activities predicted by GSVA (Figs. 1, 4 and 5). A similar phenomenon was identified in BCSA3 TumA1, where tumors were regionally separated by different clone events in chr 7 and 8 while maintaining distinct GEX cluster compositions (Supplementary Figs. 9, 13, 14). Nonetheless, no such findings were observed in BCSA2 (Supplementary Figs. 6, 12).

We also piloted studying the spatially resolved breast cancer intrinsic subtype analysis in the study. Remarkably, in comparison to other subtypes, normal-breast-like was found in all tumors and likely contributed more extensively by noncancerous components within TME. In HER2-positive BCSA2 and BCSA3 samples, luminal subtypes were also observed at different proportions, consistent with their immunohistochemistry (IHC) based biomarker status (Supplementary Figs. 1, 15, 16, 17). Intriguingly, although basal-like fractions were the major molecular subtype, we discovered a decent proportion (10.88%) of HER2-enriched areas in BCSA1, whereas no HER2 protein expression was detected by IHC (Fig. 6, Supplementary Fig. 1). Overall, we observed a mixed pattern of various subtypes within each tumor, indicating the complexity of breast cancer biology and the importance of considering the heterogeneity of molecular subtypes for therapeutic decision-making.

In a recent study, we reported the development of a new spatial transcriptomics-derived technology, namely Spatial VDJ, using frozen sections to map full-length B cell receptor and T cell receptor sequences and their lineage relationships within tissues at individual spot resolution^9^. Here, we proposed to compliment it with CTA on the paired H&E images for the enhanced definition of visualization, which enabled us to understand the genuine interaction between specific lymphocyte clones and tumor cells (Fig. 7). This is with great opportunities to understand for example, which cytotoxic T cell clones situated at the tumor invasive front for studying their specificity towards cancer immunotherapy or engineered cell therapies. Alternatively, the positional information of the specific B cell clones located at the tumor niches could indicate possible antibodies that are worth further development into therapeutics, such as anti-cancer vaccines^43^.

Despite CTA’s clear advantages in assisting ST data interpretation, we also noticed some limitations. Firstly, this method demands high-quality original images to achieve optimal performance. The accuracy of the cell type classification could be influenced by many factors including tissue quality, H&E staining, and imaging conditions. Additionally, we have only tested it on the most common ductal carcinomas but not yet on lobular cancer or other rare breast cancer subtypes. Lastly, due to the confined training material, we did not stratify our dataset into training and testing sets. To generalize our image-based object classifier, a much larger dataset is needed for model training and cross-validation, which is not available in the current study. However, we believe such limitations could be addressed, provided that adequate resources are accessible.

**Fig. 8:**
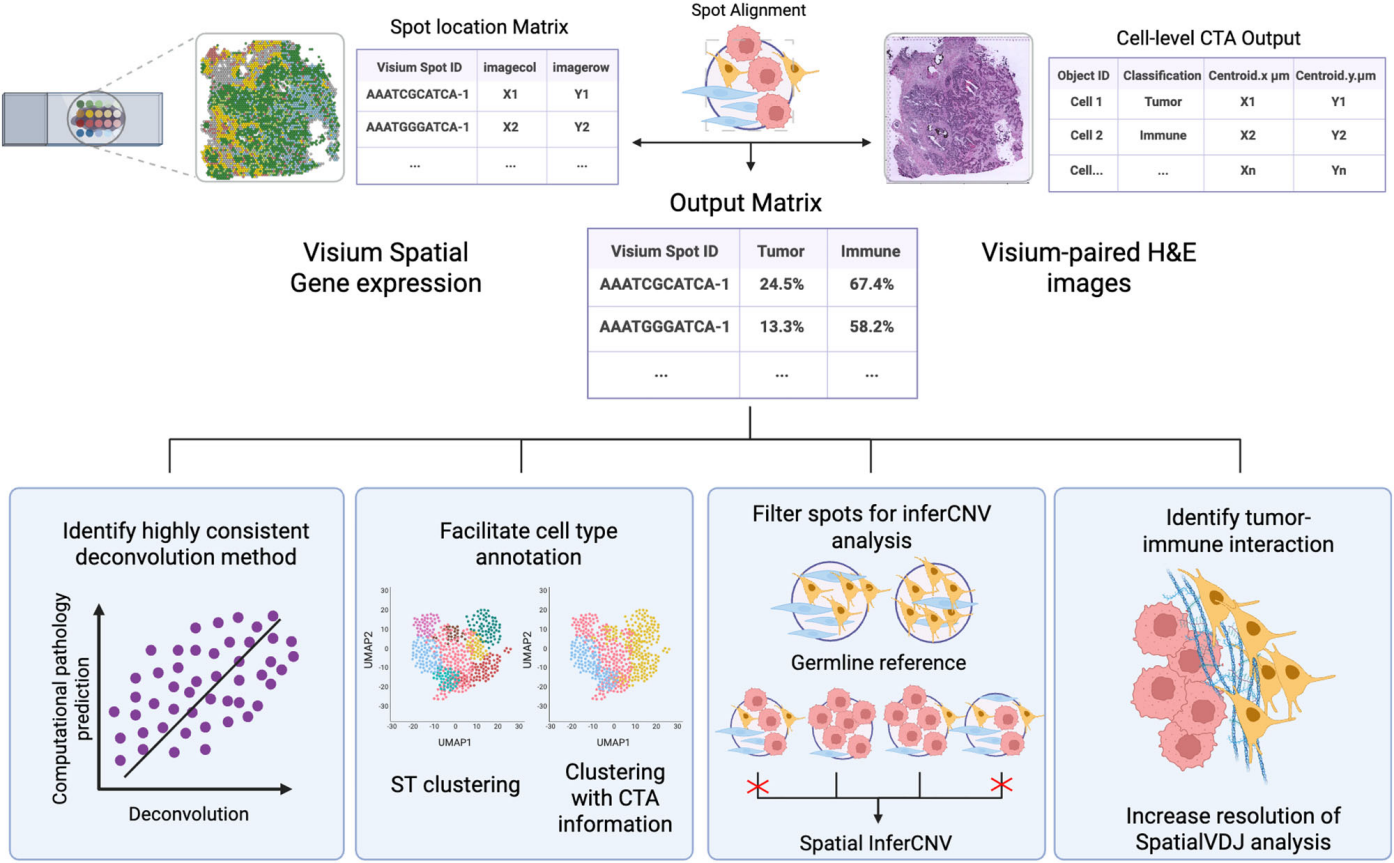
Proposed workflow for optimized spatial transcriptomic data analysis by incorporating computational tissue annotation. Combining the spatial information, we performed spot alignment on cell-level annotation obtained from the paired H&E images, resulting in an output matrix containing percentages of tumor, immune, and stroma cells in each Visium spot. In the current study, we incorporated the CTA pipeline and improved the resolution of the ST analysis. Through Spearman’s correlation test, we compared the output matrix to the results from deconvolution to identify the best-performing methods in our dataset. In addition, by plotting cell type percentage on the GEX-based clustering UMAP, CTA results could facilitate the spot-level cell type annotation. In parallel, we incorporated CTA into spatial copy number variation estimation to better define germline reference and quantitatively identify spots with a stringent cutoff of tumor percentage. Lastly, we demonstrated that by visualizing the cell annotation on the H&E images using CTA, we could increase the resolution of SpatialVDJ analysis on spots where specific immune cell clones exist. To conclude, joint analysis of ST with the CTA pipeline could improve the data interpretation and overcome current technical limitations. Created in BioRender. Li, T. (2025) https://BioRender.com/mtucnea.

In conclusion, our user-friendly CTA pipeline serves as a complementary approach that provides valuable morphological context to support GEX–based ST analysis and enhance the biological interpretation of breast cancer samples. We demonstrated its value in assessing the performance of deconvolution methods, facilitating GEX clustering annotation and copy number estimation, and improving the visualization resolution of tumor-immune cell clone interactions in our dataset (Fig. 8). Although not demonstrated in the current study, our CTA pipeline has the potential to be applied on other cancer types given the accessible materials and disease area expertise.

## Methods

### Patient recruitment and sample collections

Breast cancer samples were collected at Karolinska University Hospital, Stockholm, Sweden from patients with untreated invasive ductal carcinomas. Patients were given information about the research project and signed a written consent according to the Declaration of Helsinki. Breast cancer sample 1 (BCSA1) was a triple-negative tumor while the rest of the samples were HER2-positive. All tumor samples were assessed for the status of hormone receptors, Ki67, and HER2 expression by pathologists. For each patient, different regions (*n* = 2–5) were selected and used for downstream spatial gene expression profiling. The isolated region was washed and embedded in OCT immediately upon receipt. The embedded samples were frozen on dry ice and stored at −80 °C until further processing. The study was approved by the Regional Ethics Review Board (Etikprövningsnämnden) in Stockholm.

### Spatial transcriptomics library preparation and sequencing

Libraries for spatial transcriptomics were prepared using Visium spatial gene expression kits as described in our previous paper^9^. In brief, OCT-embedded tissues were sectioned (10 μm) and placed on the glass slides with a 6.5 mm × 6.5 mm capture area. After H&E staining and mounting with 85% glycerol, images were taken using 20X objective with the Metafer Slide Scanning platform (Microscope stand: AxioImager.Z2 with ScopeLED Illumination, Zeiss; Camera: CoolCube 4 m, MetaSystems; Objective: Plan-Apochromat 20X/0.80 M27, a = 0.55 mm, Zeiss, Software: Metafer5 version 3.14.192) as reported previously^5^. Raw images were exported using the VSlide software (version 1.1.128; MetaSystems). Subsequently, the coverslips were removed according to the protocol. Tissues were permeabilized to release the RNA which then underwent reverse transcription, second-strand synthesis, and denaturation. The obtained cDNA samples were amplified, purified, and used for library construction. After fragmentation, end-repair, and A-tail addition, adaptor and sample index were added to the purified cDNA to obtain the final libraries. Sequencing was performed using the Illumina NovaSeq 6000 or NextSeq2000 platform at the National Genomics Infrastructure, SciLifeLab in Solna, Sweden.

### Computational pathology annotations using QuPath

H&E images (in tiff or jpeg format) paired with spatial transcriptomics were imported and analyzed using QuPath (version 5.1.0). The pixel size was verified for each image and manually corrected based on the image metadata. Regions of interest were then selected using the polygon annotations. To correct the H&E-stained vector for the image analysis, an area comprising 60% of the tissue and 40% of the blank background was chosen. After excluding unrecognized colors, automated correction was performed using the default setting.

Unsupervised cell segmentation was performed with the default setting for tiff images (scale in pixel/μm) and with a customized setting for jpeg images (scale in pixel). Subsequently, the identified cell nucleus was smoothed using the calculate features and *Add smoothed features* functions in QuPath with the default and twice the default setting sequentially (25 μm and 50 μm for tiff images; 100 px and 200 px for jpeg images, respectively). Object classifier was trained interactively using the *Train object classifier* function with Random trees algorithm, incorporating all measurements and normalized by mean and variance on pre-defined cell categories (i.e., tumor, immune, and stroma cells). We manually annotated example cells and iteratively loaded them into the algorithm. Finally, the output was exported as a text file for later processing using the *show detection measurement* function. Features used for training the object classifier were provided in Supplementary Data 1.

### Data integration and analysis

RNA sequencing data was aligned and mapped against homo sapien genome GRCh38 (GRCh38-3.0.0) using SpaceRanger (version 1.0.0). We downloaded the data from Engblom et al^9^ and Llorens-Bobadilla et al.^10^, and together with our data, we included 23 sections from BCSA1, BCSA2, BCSA3, and BCSA4 samples. Sequencing data from BCSA2 Region A was removed due to low quality. Data analysis was performed using Seurat (version 5.0.3)^44,45^, Semla (version 1.1.6)^46^, STUtility (version 1.1.1)^47^, GSVA (version 1.50.5)^23^, clusterProfiler (version 4.10.1)^48,49^, Spatial inferCNV (version 0.1.0)^5^, AIMS^28^, and biomaRt (version 2.58.2)^50,51^ through R (version 4.3.2) and RStudio (version 2023.12.0 + 369). Low-quality spots with less than 500 detected genes, more than 25% of mitochondrial genes, and 20% of hemoglobulin genes were removed from the downstream analysis. In addition, mitochondrial genes were removed from the count matrix. Data from the same patient was merged, log-normalized, and scaled before rPCA integration using the top 2000 variable genes. Subsequently, the percentage of mitochondria was regressed out during data scaling. NMF analysis was performed using the STUtility package. We used 20 NMF factors for BCSA1, BCSA2, and BCSA3 samples with a resolution of 0.4, 0.55, and 0.4, respectively. Differential expression analysis was performed using the *FindAllMarkers* function with the Wilcox test.

### Gene set variation analysis (GSVA)

GSVA was performed to investigate the differences among different tumor clusters. A pseudo-bulk count matrix was obtained by applying the *AggregateExpression* function to each cluster. After CPM (counts per million) normalization and log transformation, we used the *gsvaParam* function to calculate the enriched pathway activities of each cluster against the hallmark gene set^22^. Visualization was performed using the ComplexHeatmap (version 2.18.0) package^52,53^ in RStudio.

### Breast cancer intrinsic subtype estimation

For molecular intrinsic subtype estimation, the Absolute Intrinsic Molecular Subtyping (AIMS), a single sample prediction based method was employed, as described by Paquet and Hallett^28^. Specific inclusion criteria were applied to ensure the reliability of subtype assignments by excluding spots consisting solely of non-malignant cells. Only spots that passed initial quality control checks, exhibited at least 20% epithelial content (tumor or normal, as determined by Cell2location deconvolution), and contained at least one tumor cell (identified through computational tissue annotation) were included.

### Copy number variation estimation with Spatial inferCNV

The copy number variation of each sample was estimated using the Spatial InferCNV^5^ (version 0.1.0) and inferCNV package^54^ (version 1.18.1). We first combined spots from different sections for the same patient. Only the first technical replicate of each region was included. Subsequently, spots without tumor cell counts were identified and used as normal cell reference based on cell-level CTA. Furthermore, we filtered out spots with less than 50%, 70%, and 90% of tumors according to annotation. The rest of the spots were included in the CNV analysis using *infercnv::run* function with analysis mode “subcluster”, HMM report by cell, and set TRUE for cluster_by_groups and denoise arguments. The scaled output was then clustered using the Ward.D method and the number of clones was determined based on hierarchical clustering. Dendrograms were cut for BCSA1 (50%: 153; 70%: 132; 90%: 110), BCSA2 (50%: 1594; 70%: 1380; 90%: 1036), and BCSA3 (50%: 255; 70%: 297; 90%: 176), respectively, using the *cutree* function.

### Deconvolution

The reference single-cell RNA sequencing (scRNAseq) data were downloaded from GSE176078^12^. We subsetted the reference data to include only triple-negative breast cancer (TNBC) samples and HER2-positive samples. Subsequently, 1000 cells were randomly selected in each cell type in the minor category (29 cell types in total) for later deconvolution. We used the same cells from the scRNAseq data for 7 deconvolution methods including CARD (version 1.1)^15^, Cell2location (version 0.1.4)^16^, RCTD (spacexr, version 2.2.1)^17^, Stereoscope (version 0.3.1)^18^, CytoSPACE (version 1.1.0)^19^, SpatialDWLS (version 4.1.6)^20^, and Tangram (version 1.0.4)^21^. Only genes shared between scRNAseq and spatial transcriptomic data were kept for the analysis. Additionally, we filtered out low-quality spots with less than 500 detected genes, more than 25% of mitochondrial genes, and more than 20% of hemoglobin genes. For deconvolution methods that incorporates internal algorithms for marker selection (i.e., CARD, Cell2location, RCTD, CytoSPACE, and SpatialDWLS), default settings were used as these tools were optimized based on their built-in selection strategies. For Stereoscope and Tangram, where an input of a predefined marker list was required, we used a marker panel identified in our previous study^9^.

CARD deconvolution was performed using the minor category of the cell type in scRNAseq data with spots in spatial transcriptomics harboring at least 5 counts and genes with at least 100 counts using the unnormalized matrix.

Stereoscope deconvolution was performed using the unnormalized count matrix with the subset scRNAseq reference. We used the previously identified highly expressed genes and cell type markers^9^ for both TNBC and HER2-positive samples. Epochs 50,000 and batch size 2048 for spatial and single-cell data with a learning rate of 0.01 were applied in the analysis.

CytoSPACE was applied to the unnormalized dataset with the default setting according to the tutorial.

SpatialDWLS deconvolution was performed using Giotto. The normalized mean gene expression of each cell type was used as the reference created by the *makeSignMatrixDWLSfromMatrix* function. The ST data was normalized using a scale factor of 10000, and highly variable features were calculated using the *calculateHVF* function. Deconvolution was performed through the *runDWLSDeconv* function with SpaceRanger clusters, n_cell = 50 for BCSA1 and 20 for the rest of the samples.

RCTD was performed using the package spacexr. Analysis was run on the object created with *create.RCTD* function (CELL_MIN_INSTANCE equals 2) using *run.RCTD* with default parameters and doublet_mode as “full”. After that, the raw output of RCTD was normalized by row to calculate the percentage of each cell type.

Cell2location deconvolution was performed according to the tutorial on the unnormalized data. In brief, reference signatures were estimated using highly variable genes selected using the *filter_genes* function with cell_count_cutoff = 5, cell_percentage_cutoff2 = 0.03, and nonz_mean_cutoff = 1.12. Then, using a negative binomial regression model, we estimated the signatures using *cell2location.models.RegressionModel.setup_anndata* function, accounting for sample batch effects and using the minor cell type. Subsequently, the regression model was trained using max_epochs = 250, and the mean expressions of selected genes per cell type were used for downstream mapping. We subsetted the reference and spatial data to only include shared genes. The model was created using *cell2location.models. Cell2location* function with N_cells_per_location = 50 for BCSA1, 20 for the rest of the samples, and detection_alpha = 20. After this, the created model was trained using the *mod. train* function with max_epochs = 30,000, batch_size = None, and train_size = 1. The “q05_cell_abundance_w_sf” column, representing the 5th percentile of the posterior distribution of cell type abundances, was saved and normalized by row to obtain the cell type abundances in percentage.

Tangram deconvolution was performed using the same features as stated above^9^. *Map_cells_to_space* function was applied to unnormalized data with cell mode and rna_count_based for density prior argument. Subsequently, annotations from scRNAseq reference (cell type minor) were projected to the spatial data with the *project_cell_annotations* function.

### Correlation between computational pathology classification and RNAseq deconvolution

To compare results from computational pathology classification and deconvolution, we first aligned the annotation to the Visium spots. One sample was excluded due to the mismatch of image metadata.

The full-size image coordinates and the radius for Visium spots were calculated using a scaling factor since the 30% down-sized images (30p images) were used to run the SpaceRanger analysis. The scaling factor was determined by dividing the height of 30p images by the height of the full-size image. As the images used in the SpaceRanger analysis had their y-axis reversed, the actual y coordinates were calculated by subtracting each y-coordinate from the height of the full-size image. Subsequently, the cells whose centroid coordinates fell into the square area (Visium spots’ central coordinates ± radius) were assigned the ID of the specific spot. The number of each cell type (i.e., tumor, immune, and stroma cells) was calculated in each spot and the percentage was determined by dividing the number of cells by the total cell count in each position.

Furthermore, to match the cell type categories in deconvolution with the computational pathology annotation, we summed up the percentage of each cell type in deconvolution to the tumor, stroma, and immune cells using the classification defined in Wu et al.^12^. After this, we performed Spearman’s correlation analysis to investigate the correlation between the results from computational pathology and deconvolution. The performance of each deconvolution method was assessed using the Spearman correlation coefficient values. Kruskal–Wallis with Dunn’s test and Benjamini–Hochberg’s correction was applied to study the difference among 7 methods and the significance of the comparison between each technique and Cell2location was marked in the Violin plot. Visualization was achieved using ggplot2 and Seurat in RStudio.

For the comparison between Xenium data and CTA outcomes, the scaling factors were manually calculated based on the image coordinates before spot alignments. Subsequently, we plotted the expression of cell-type-specific markers (*ERBB2*-tumor, *PTPRC*-immune, and *PDGFRA*-stroma) and compared them to the CTA results. As the Xenium platform mainly measured the number of transcripts that were differentially expressed in different cell types, we plotted 15%, 50%, and 100% of *ERBB2*, *PTPRC*, and *PDGFRA* transcripts, respectively, to match the number of cells identified in CTA.

## Supplementary information

Supplementary Data 1

Supplementary Data 2

Supplementary Information

## Data Availability

The raw sequencing data have been deposited in the Swedish National Data Service (DORIS) with the DOI number 10.48723/f4v5-m008. The processed data from the corresponding samples were deposited in Zenodo with the DOI number 10.5281/zenodo.15211538. Parts of the raw sequencing data were downloaded from the European Genome-phenome Archive (EGA), which is hosted by the EBI and the CRG with the accession numbers EGAD00001011061. The processed data from BCSA2 and BCSA3 were downloaded from Zenodo 10.5281/zenodo.7961605 and 10.5281/zenodo.10255434. For BCSA4, the processed data were available at Gene Expression Omnibus with the accession code GSE214991. Xenium data used for computational tissue annotation and analysis was available at https://www.10xgenomics.com/products/xenium-in-situ/preview-dataset-human-breast. Codes for analysis are available at https://github.com/JohanHartmanGroupBioteam/BreastCancer_CTA.
