# Supplementary material for: Computational pathology annotation enhances the resolution and interpretation of breast cancer spatial transcriptomics data
Source: NPJ Precis Oncol. 2025 Sep 9;9:310. doi: 10.1038/s41698-025-01104-3 (PMC12420830; doi:10.1038/s41698-025-01104-3)
Supplement: Supplementary file 3 — Supplementary Information [file 41698_2025_1104_MOESM3_ESM.pdf]

Computational pathology annotation enhances the resolution and interpretation of breast cancer spatial transcriptomics data

Tianyi Li [1,#], Qiao Yang [1], Balazs Acs [1,2], Emmanouil G. Sifakis [1], Hosein Toosi [3], Camilla Engblom [4,5], Kim Thrane [6], Qirong Lin [4], Jeff E. Mold [4], Wenwen Sun [1,2], Ceren Boyaci [1,2], Sanna Steen [1,2], Jonas Frisén [4], Jens Lagergren [3], Joakim Lundeberg [6], Xinsong Chen [1,7,#], Johan Hartman [1,2,7]

1 Department of Oncology and Pathology, Karolinska Institutet, Stockholm, Sweden.

2 Department of Clinical Pathology and Cancer Diagnostics, Karolinska University Hospital, Stockholm, Sweden.

3 SciLifeLab, Department of Computation Science and Technology, KTH Royal Institute of Technology, Stockholm, Sweden.

4 Department of Cell and Molecular Biology, Karolinska Institutet, Stockholm, Sweden.

5 SciLifeLab, Division of Immunology and Respiratory Medicine, Department of Medicine Solna, Karolinska Institutet, Center for Molecular Medicine, Karolinska University Hospital.

6 SciLifeLab, Department of Gene Technology, KTH Royal Institute of Technology, Stockholm, Sweden.

7 These authors contributed equally to this work.

# Corresponding author: Xinsong Chen, xinsong.chen@ki.se; Tianyi Li, tianyi.li.2@ki.se

Supplementary

Supplementary Figure 1

Overview of the human breast cancer samples (BCSA).

Supplementary Figure 2

Comparison between Xenium and computational tissue annotation (CTA).

Supplementary Figure 3

Spatial localization and expression of immune, stroma, and tumor markers.

Supplementary Figure 4

Heatmap of top 15 differentially expressed genes (DEGs) ranked by log2 fold change in each cluster within BCSA1 sample.

Supplementary Figure 5

Analysis of two tumor regions from the TNBC sample identifies different tumor clusters at gene expression level.

Supplementary Figure 6

Analysis of HER2-positive samples (BCSA2) identifies transcriptional intra-tumoral heterogeneity patterns.

Supplementary Figure 7

Heatmap of top 15 differentially expressed genes (DEGs) ranked by log2 fold change in each cluster within BCSA2 sample.

Supplementary Figure 8

Analysis of four tumor regions from the HER2-positive sample (BCSA2) identifies different tumor clusters at gene expression level.

Supplementary Figure 9

Analysis of HER2-positive samples (BCSA3) identifies transcriptional intra-tumoral heterogeneity patterns.

Supplementary Figure 10

Heatmap of top 15 differentially expressed genes (DEGs) ranked by log2 fold change in each cluster within BCSA3 sample.

Supplementary Figure 11

Analysis of four tumor regions from the HER2-positive sample (BCSA3) identifies different tumor clusters at gene expression level.

Supplementary Figure 12

Spatial determination of CNV status to identify clonal events in HER2-positive tumor (BCSA2).

Supplementary Figure 13

Spatial determination of CNV status to identify clonal events in HER2-positive tumor (BCSA3).

Supplementary Figure 14

Sankey plots showing the distribution of gene expression clusters by inferred clones.

Supplementary Figure 15

Heterogeneity of spatially resolved breast cancer intrinsic subtypes in HER2-positive tumor (BCSA2).

Supplementary Figure 16

Heterogeneity of spatially resolved breast cancer intrinsic subtypes in HER2-positive tumor (BCSA3).

Supplementary Figure 17

Spatial localization and expression of breast cancer-specific biomarkers.

Supplementary Data 1

List of features used in the training of the object classifier. The excel data sheet 1 displayed a list of features used in the training of a classifier for BCSA1, and data sheet 2 showed the list of features used for BCSA2-4.

Supplementary Data 2

The cell type compositions predicted by Cell2Location deconvolution and computational tissue annotation (CTA) for the selected spots with B and T cell clones. The Excel data sheet 1 showed the cell type compositions in spots with B cell clone 0 in the BCSA2TumE2 section, while the data sheet 2 displayed the composition for T cell clone 61 in BCSA3TumA1. The compositions are represented in percentage.

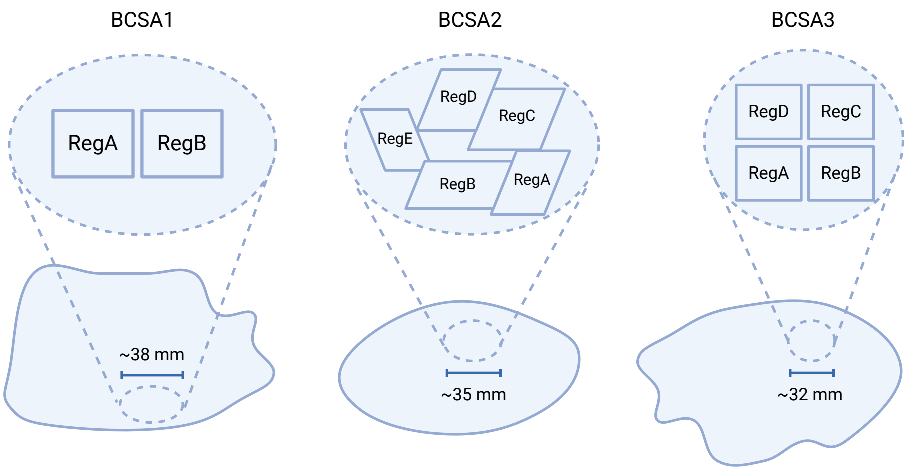

Patients	Subtype	Tumor size	Grade	Biomarker status	Samples#
BCSA1	TNBC	38 mm	NHG 3	ER 0%, PR 0%, Ki67=85%, HER2 0	Tum A1, A2, B1, B2
BCSA2	LB/HER2+	35 mm	NHG 3	ER 30%, PR 0%, Ki67=79%, HER2 3+	Tum B1, B2, C1, C2, D1, D2, D3, D4, E1, E2 *
BCSA3	LB/HER2+	32 mm	NHG 3	ER 90%, PR 90%, Ki67=70%, HER2 3+	Tum A1, A2, B1, B2, C1, C2, D1, D2
BCSA4	HER2+	30 mm	NHG 3	ER 0%, PR 0%, Ki67=40%, HER2 3+	Tum A1

#Number in the sample code indicates number of consecutive sections
*Tum A1, A2 were removed in the downstream analysis due to low quality of sequencing data

Supplementary Figure 1. Overview of the human breast cancer samples (BCSA). The images above illustrate the regional biopsies of each patient. The table below presents the clinical diagnostics of the tumors, including subtype, tumor size, tumor grade, IHC-based biomarker status, and sample index.

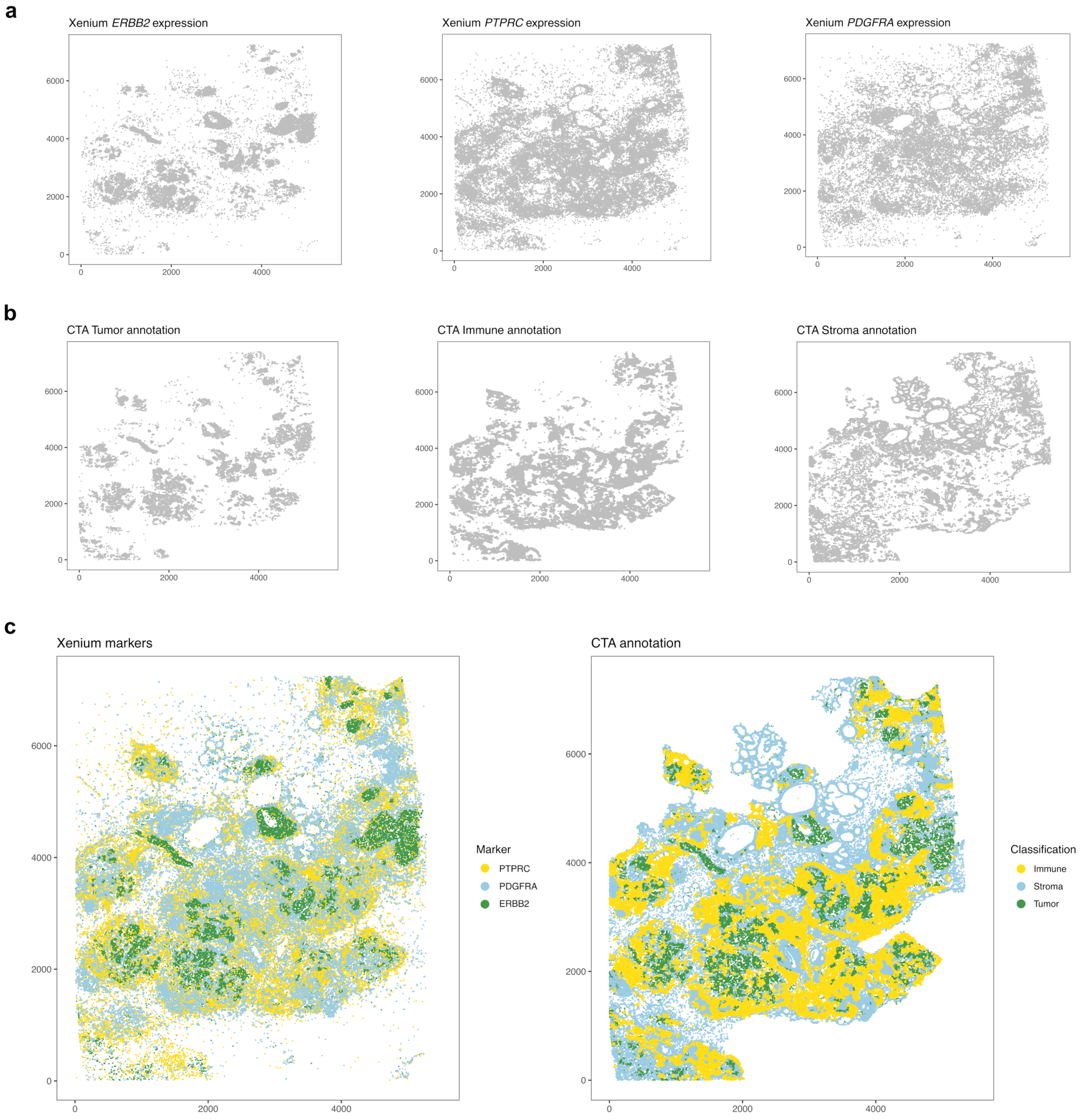

Supplementary Figure 2. Comparison between Xenium and computational tissue annotation (CTA). (a) Localization of tumor (*ERBB2*), immune (*PTPRC*), and stroma (*PDGFRA*) markers on an FFPE HER2-positive tumor sample sequenced with Xenium platform[1]. (b) Localization of tumor, immune, and stroma cells on the paired H&E image according to cell-level CTA. (c) Localization of three cell-type markers expression on the Xenium dataset (left) and annotations from CTA on the corresponding cell types (right). Yellow shows the location of the immune marker *PTPRC* and immune cells; the stroma and *PDGFRA* are indicated in blue; and the tumor and *ERBB2* are shown in green.

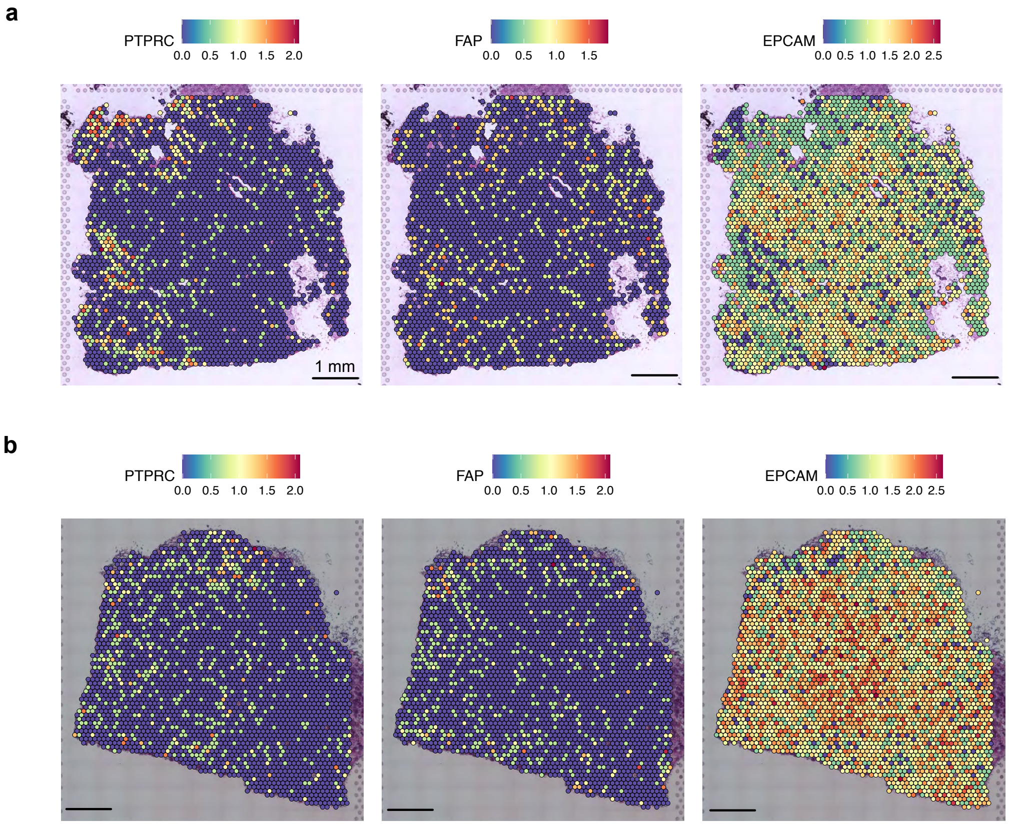

Supplementary Figure 3. Spatial localization and expression of immune, stroma, and tumor markers. (a) Expressions of immune (*PTPRC*), stroma (*FAP*), and tumor (*EPCAM*) markers on BCSA1TumA1 sample. (b) Expressions of three cell-type markers on BCSA2TumE2 sample. The color bar indicates the expression level. Scale bar 1 mm.

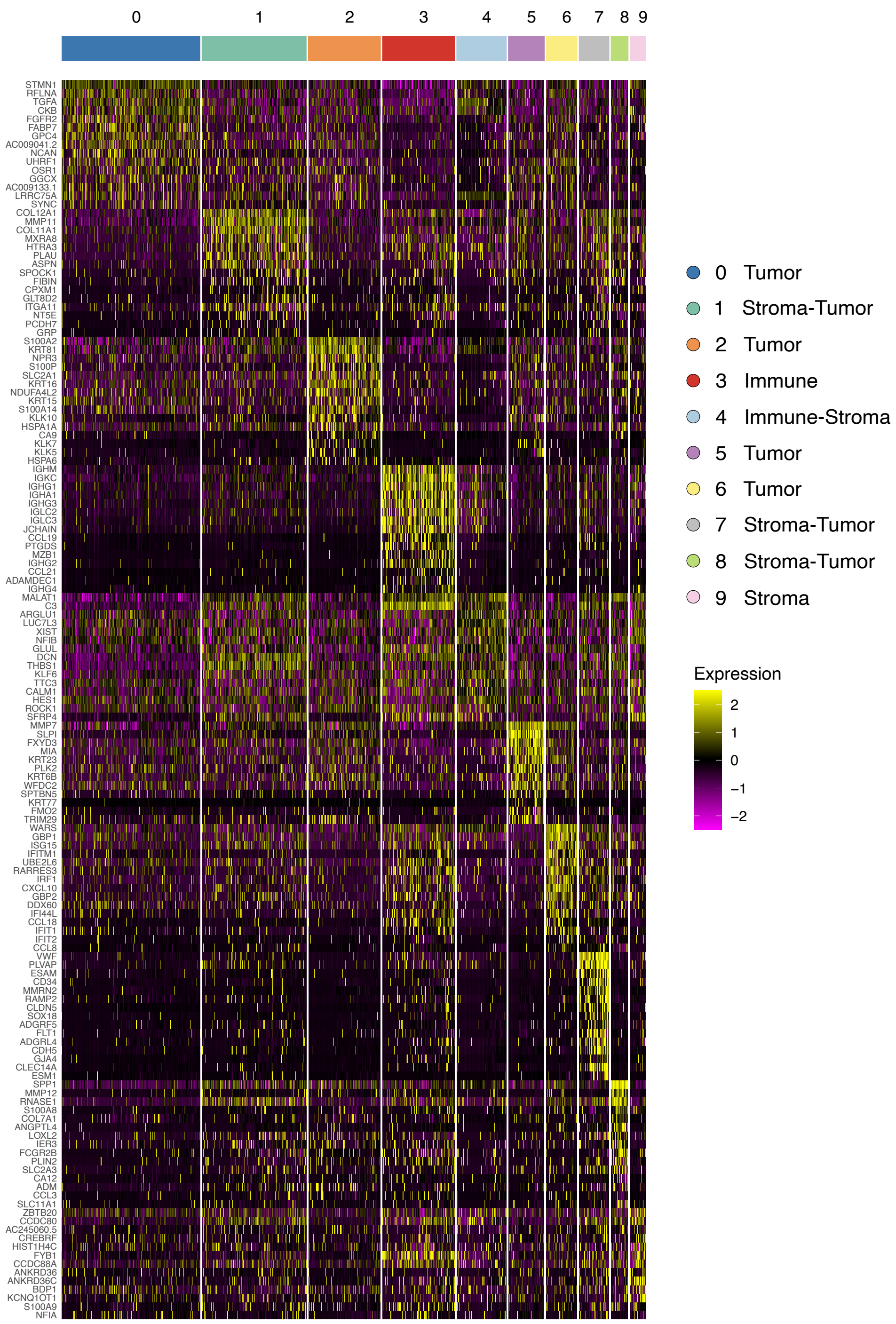

Supplementary Figure 4. Heatmap of top 15 differentially expressed genes (DEGs) ranked by log_2 fold change in each cluster within BCSA1 sample. The color indicates scaled expression levels, with purple representing low and yellow representing high expression. The top color codes represent individual gene expression clusters.

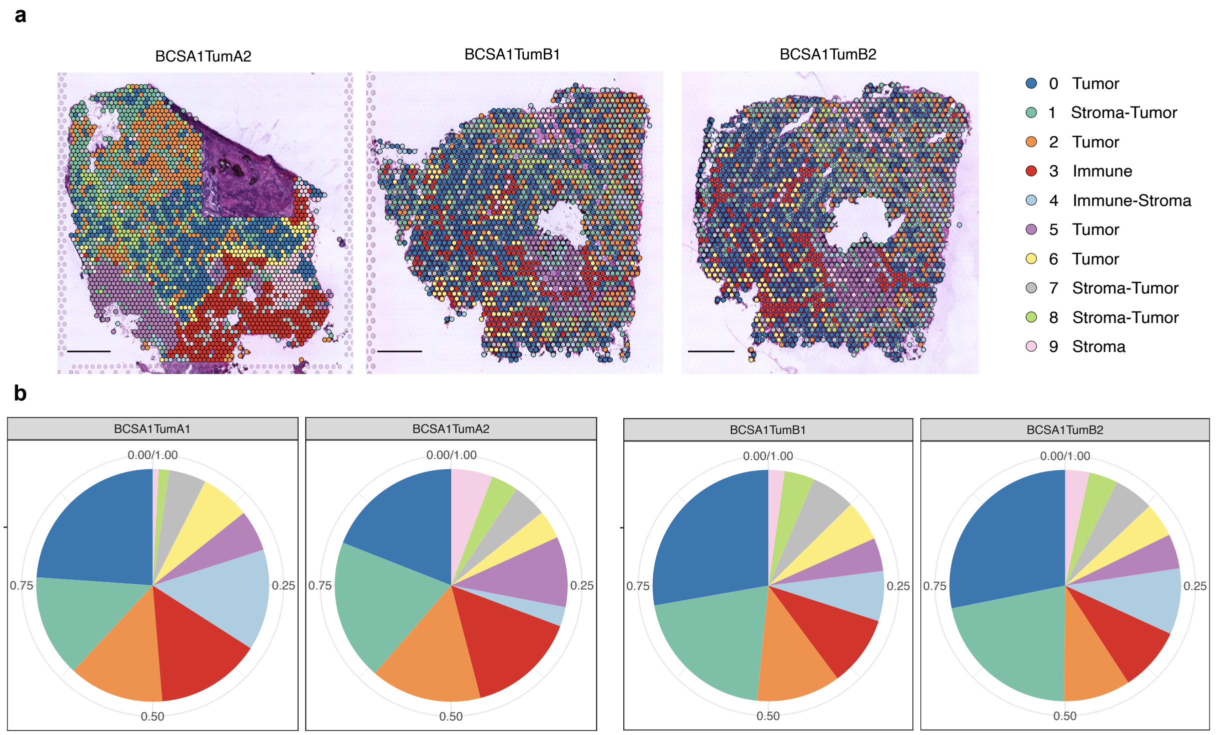

Supplementary Figure 5. Analysis of two tumor regions from the TNBC sample identifies different tumor clusters at gene expression level. (a) Spatial GEX clusters are shown on the BCSA1TumA2, TumB1, and TumB2 sections after rPCA integration of samples from areas A and B in BCSA1. Scale bar 1 mm. (b) Percentage of GEX clusters in different tumor regions. The folded tissue area in BCSA1TumA2 was excluded from the analysis.

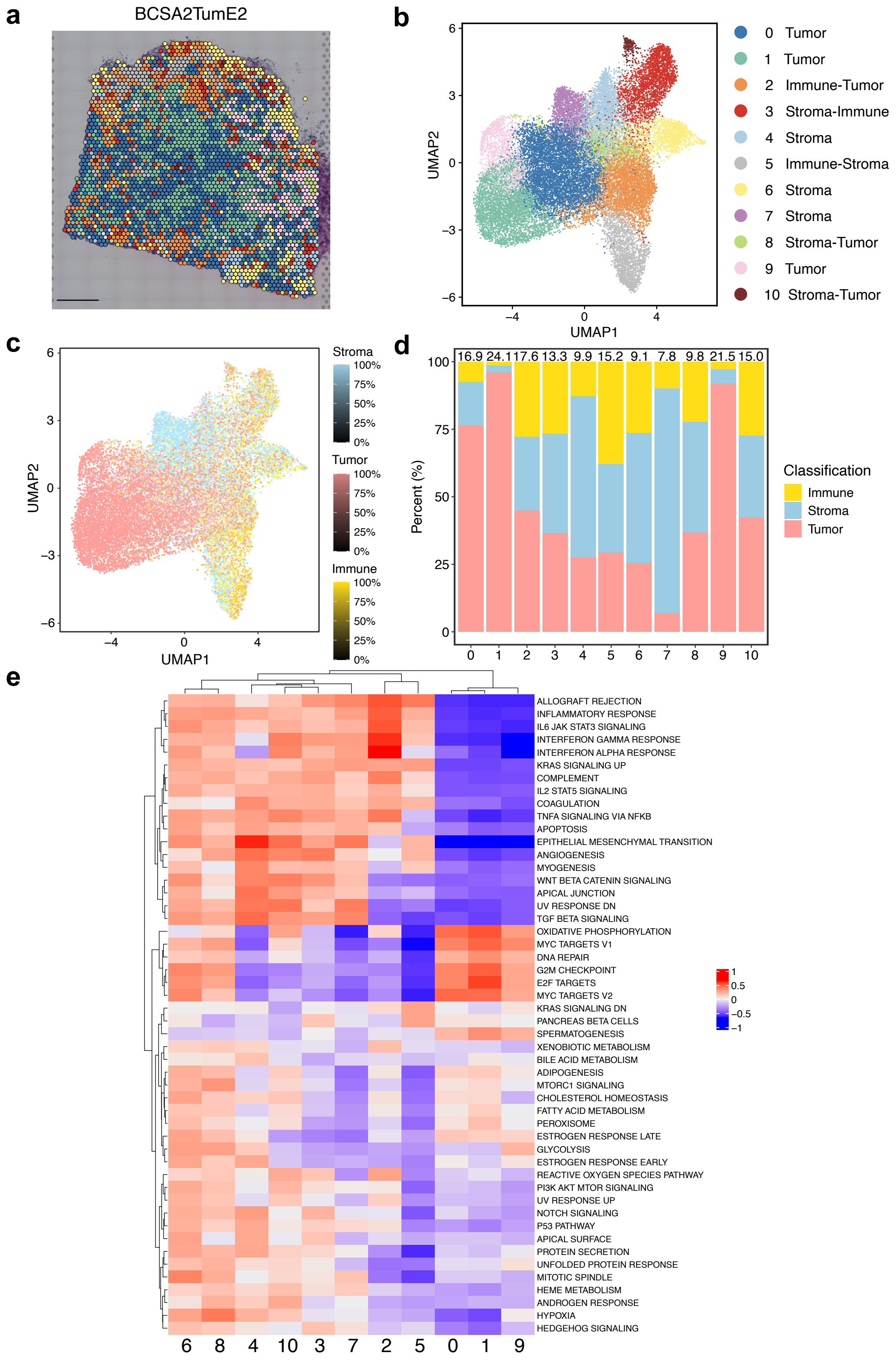

Supplementary Figure 6. Analysis of HER2-positive samples (BCSA2) identifies transcriptional intra-tumoral heterogeneity patterns. (a) Spatial GEX clusters are shown on the BCSA2TumE2 section after the rPCA integration of samples from regions B, C, D, and E.

Scale bar 1mm. (b) Uniform manifold approximation and projection (UMAP) of identified GEX clusters after integrating data from all 10 sections from regions B, C, D, and E. The color codes represent different clusters and are shown as the sidebar. (c) UMAP of the BCSA2 sample colored by the percentage of stroma, tumor, and immune cells classified by CTA. (d) Percentage of immune, stroma, and tumor predicted by CTA for each GEX cluster within BCSA2 samples. Immune cells are shown in yellow, stroma cells in blue, and tumor cells in pink. The spot density, calculated by the total number of cells/nuclei annotated by CTA divided by the number of spots in each cluster, was displayed on top of each stacked bar (cells/spot). (e) Gene set variation analysis (GSVA) on aggregated, cpm-normalized, and log-transformed count matrix in each cluster against the hallmark gene sets. The color scale indicates pathway activity predicted by GSVA, where blue represents low and red indicates high activity.

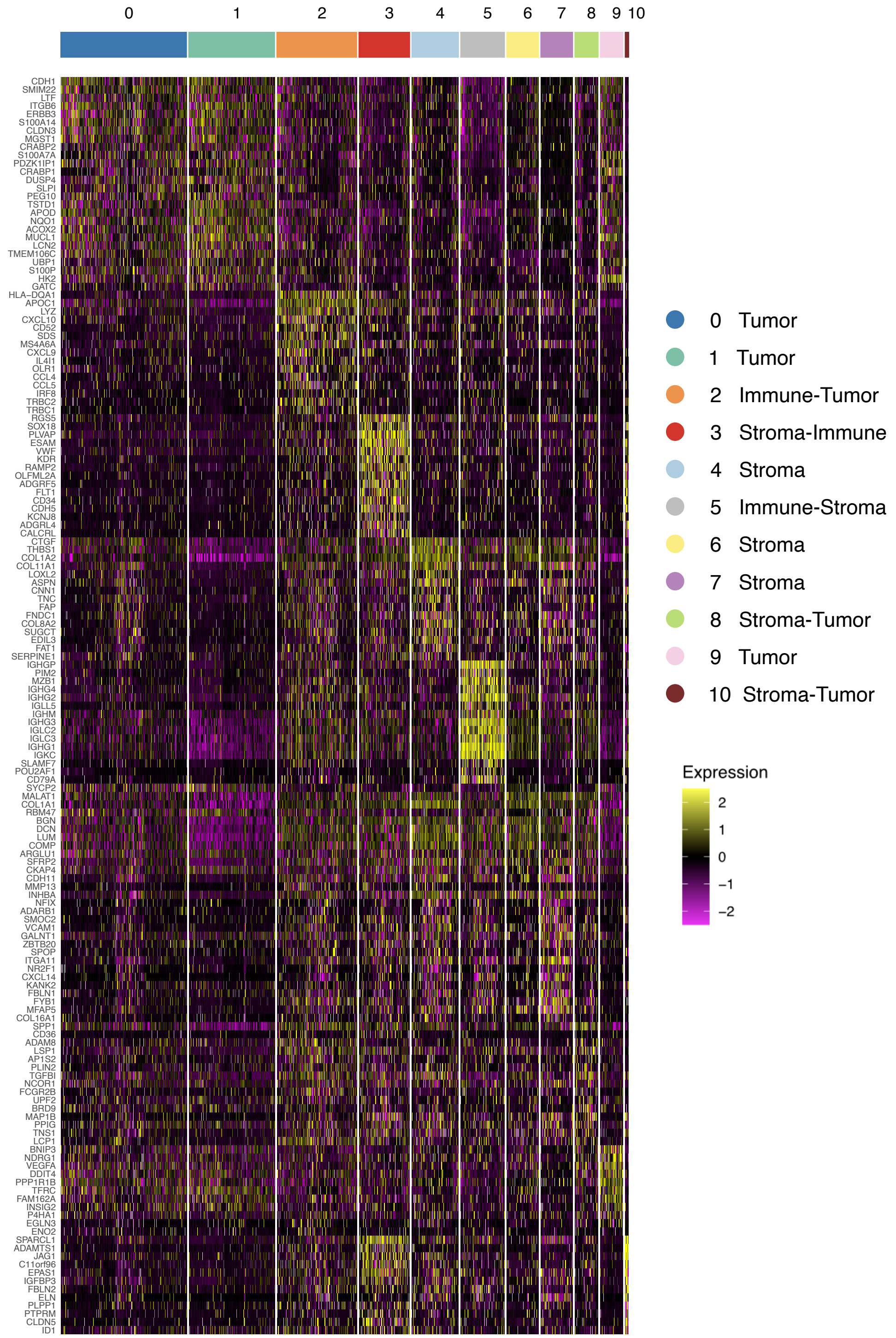

Supplementary Figure 7. Heatmap of top 15 differentially expressed genes (DEGs) ranked by $\log_2$ fold change in each cluster within BCSA2 sample. The color indicates scaled expression levels, with purple representing low and yellow representing high expression. The top color codes represent individual gene expression clusters.

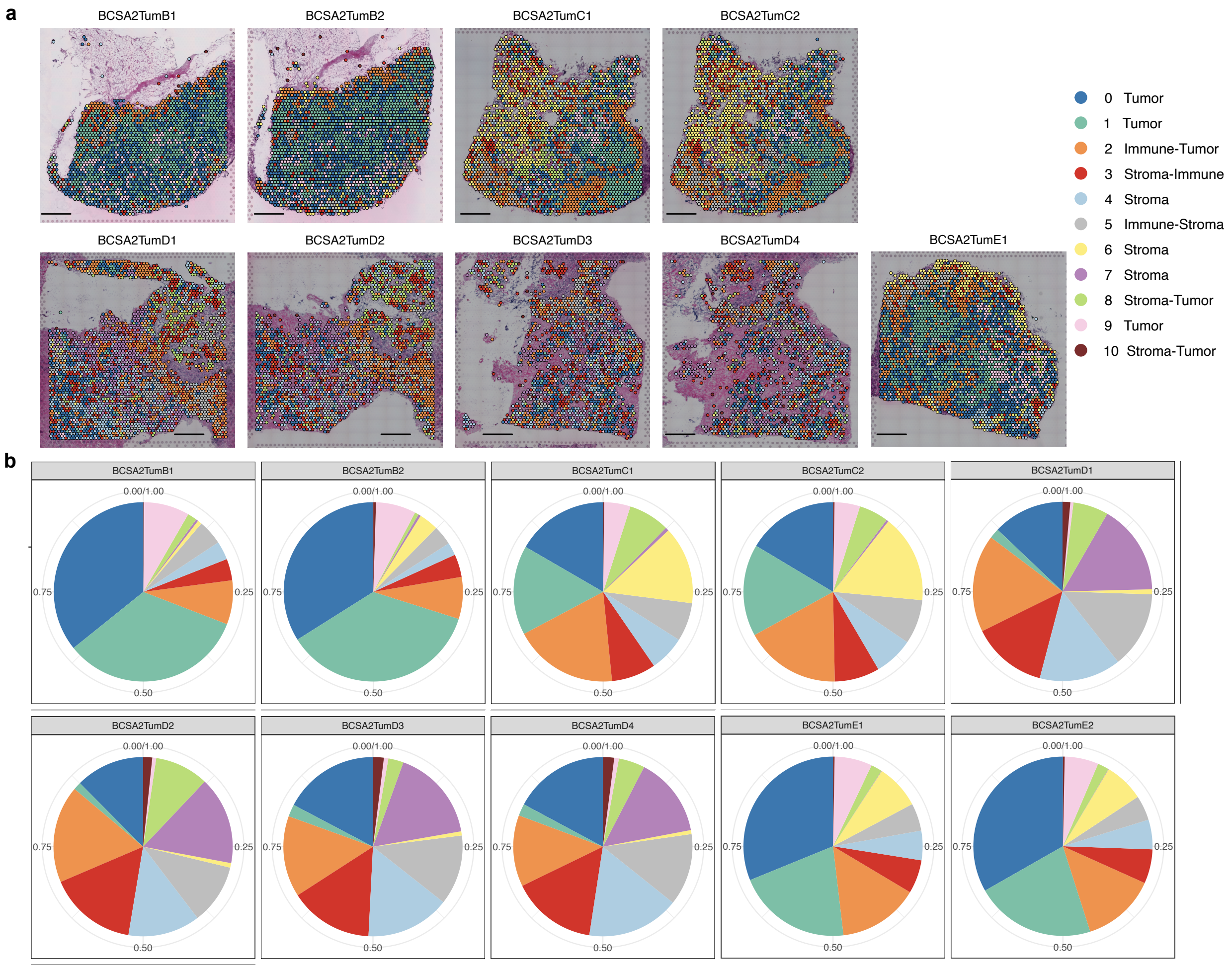

Supplementary Figure 8. Analysis of four tumor regions from the HER2-positive sample (BCSA2) identifies different tumor clusters at gene expression level. (a) Spatial GEX clusters are shown on the BCSA2TumB1, TumB2, TumC1, TumC2, TumD1, TumD2, TumD3, TumD4, and TumE1 sections after rPCA integration of samples from areas A, B, C, D, E in BCSA2. Scale bar 1 mm. (b) Percentage of GEX clusters in different tumor regions.

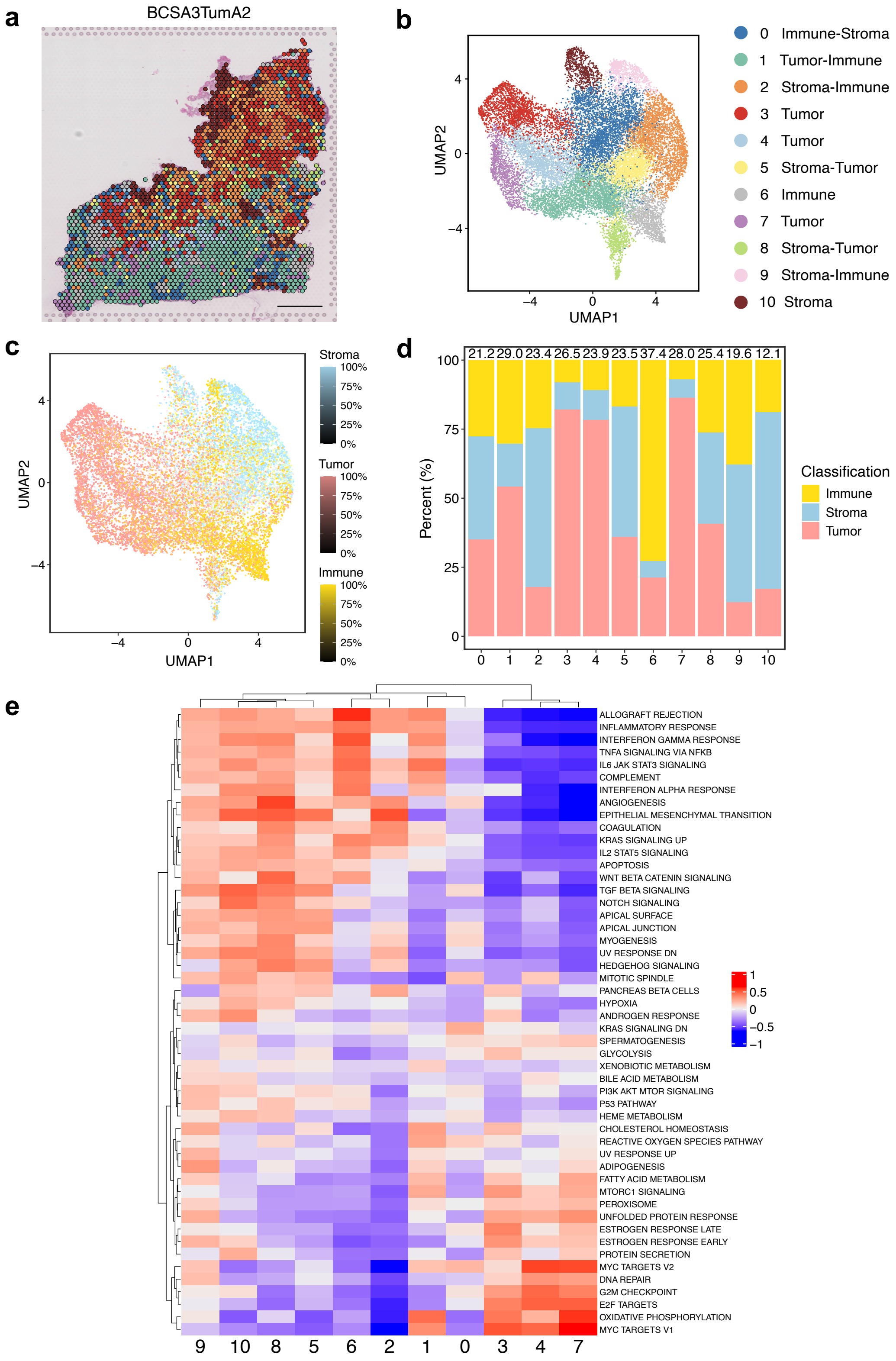

Supplementary Figure 9. Analysis of HER2-positive samples (BCSA3) identifies transcriptional intra-tumoral heterogeneity patterns. (a) Spatial GEX clusters are shown on the BCSA3TumA2 section after the rPCA integration of samples from regions A, B, C, and D.

Scale bar 1mm. (b) Uniform manifold approximation and projection (UMAP) of identified GEX clusters after integrating data from all 8 sections from regions A, B, C, and D. The color codes representing different clusters are shown as the sidebar. (c) UMAP of the BCSA3 sample colored by the percentage of stroma, tumor, and immune classified by CTA. (d) Percentage of immune, stroma, and tumor predicted by CTA for each GEX cluster within BCSA3 samples. Immune cells are shown in yellow, stroma cells in blue, and tumor cells in pink. The spot density, calculated by the total number of cells/nuclei annotated by CTA divided by the number of spots in each cluster, was displayed on top of each stacked bar (cells/spot). (e) Gene set variation analysis (GSVA) on aggregated, cpm-normalized, and log-transformed count matrix in each cluster against the hallmark gene sets. The color scale indicates pathway activity predicted by GSVA, where blue represents low and red indicates high activity.

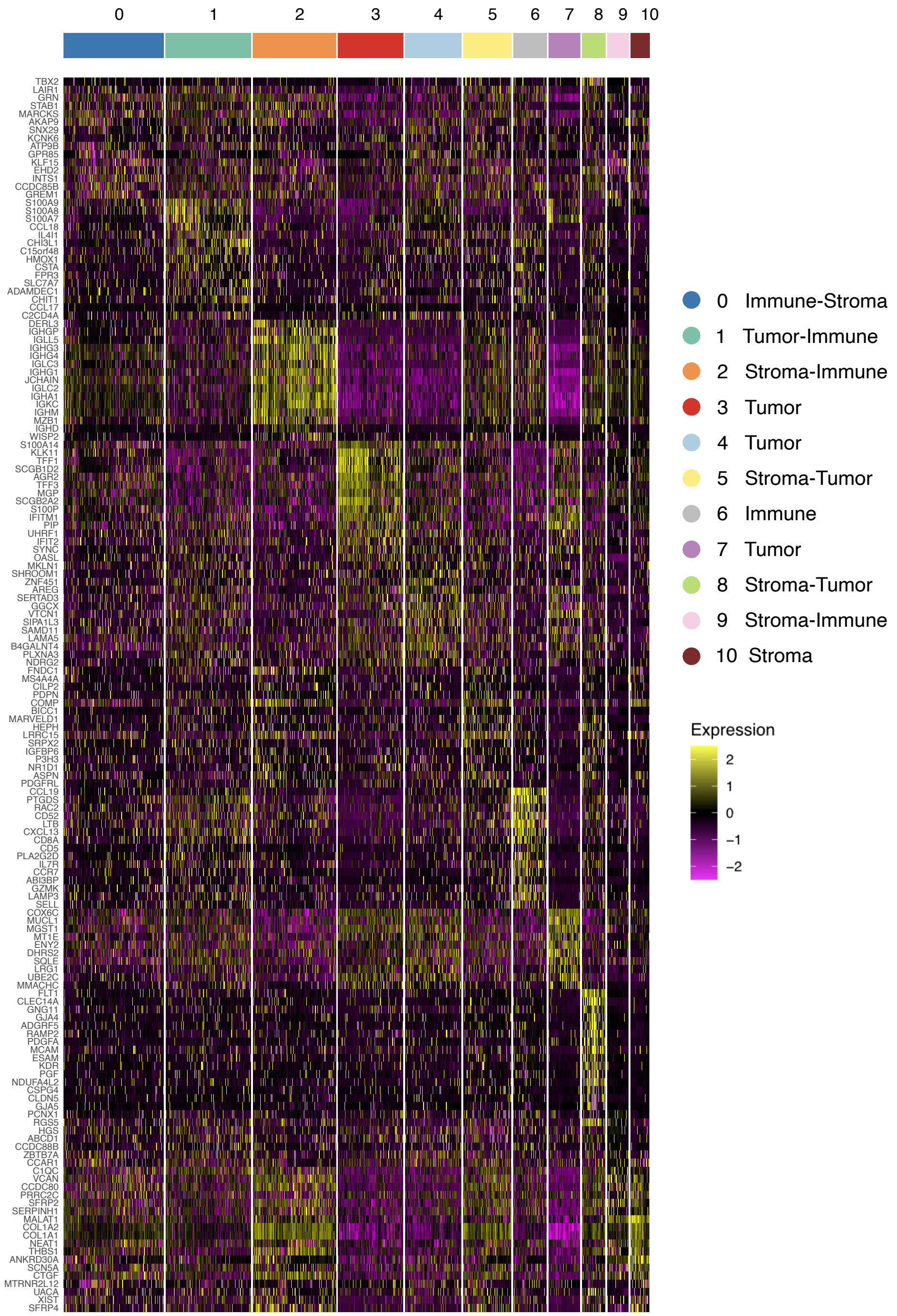

Supplementary Figure 10. Heatmap of top 15 differentially expressed genes (DEGs) ranked by $\log_2$ fold change in each cluster within BCSA3 sample. The color indicates scaled expression levels, with purple representing low and yellow representing high expression. The top color codes represent individual gene expression clusters.

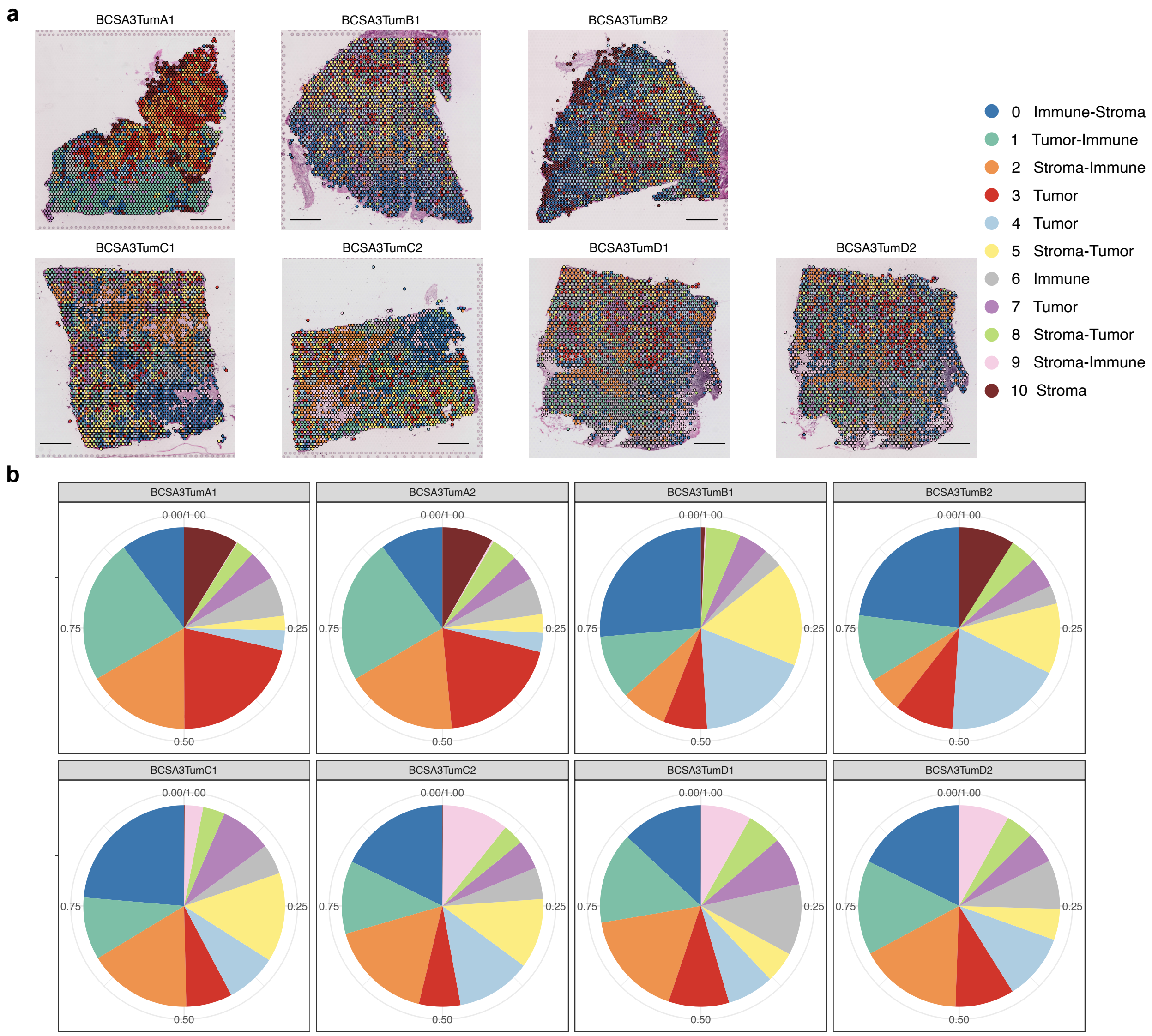

Supplementary Figure 11. Analysis of four tumor regions from the HER2-positive sample (BCSA3) identifies different tumor clusters at gene expression level. (a) Spatial GEX clusters are shown on the BCSA2TumA1, TumB1, TumB2, TumC1, TumC2, TumD1, and TumD2 sections after rPCA integration of samples from areas A, B, C, and D in BCSA3. Scale bar 1 mm. (b) Percentage of GEX clusters in different tumor regions.

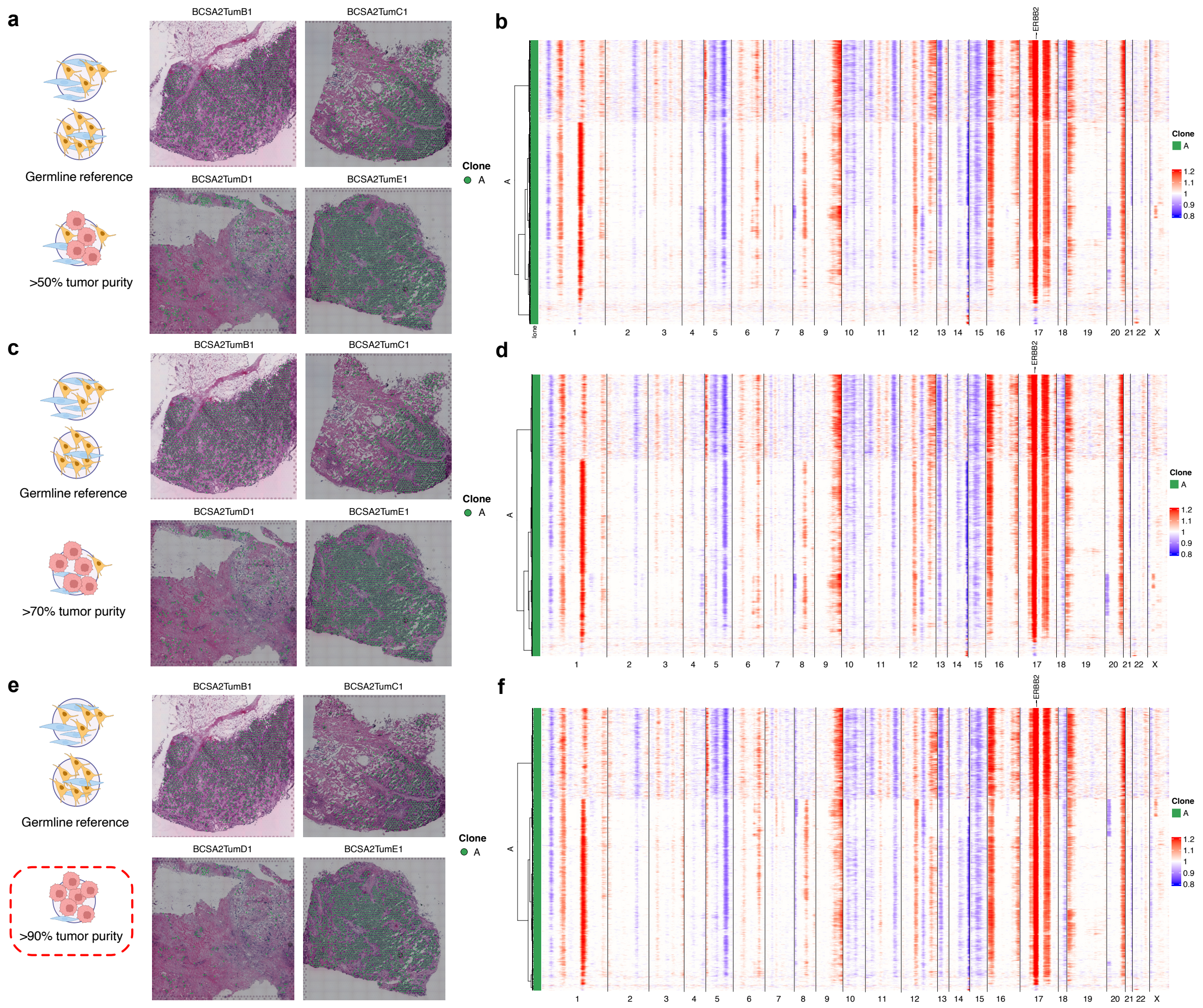

Supplementary Figure 12. Spatial determination of CNV status to identify clonal events in HER2-positive tumor (BCSA2). Spatial visualization of tumor clone on the BCSA2 TumB1, TumC1, TumD1, and TumE1 sections using spots with 50% (a), 70% (c), and 90% (e) of tumor purity defined by CTA. Genome-wide CNV analysis was performed for each spot with at least 50% (b), 70% (d), and 90% (f) of tumor cell fractions. The color indicates scaled expression levels, with blue representing copy number loss and red indicating copy number gain. The clonal grouping of spots was defined by hierarchical clustering. The chromosome numbers are displayed at the bottom of the heatmap.

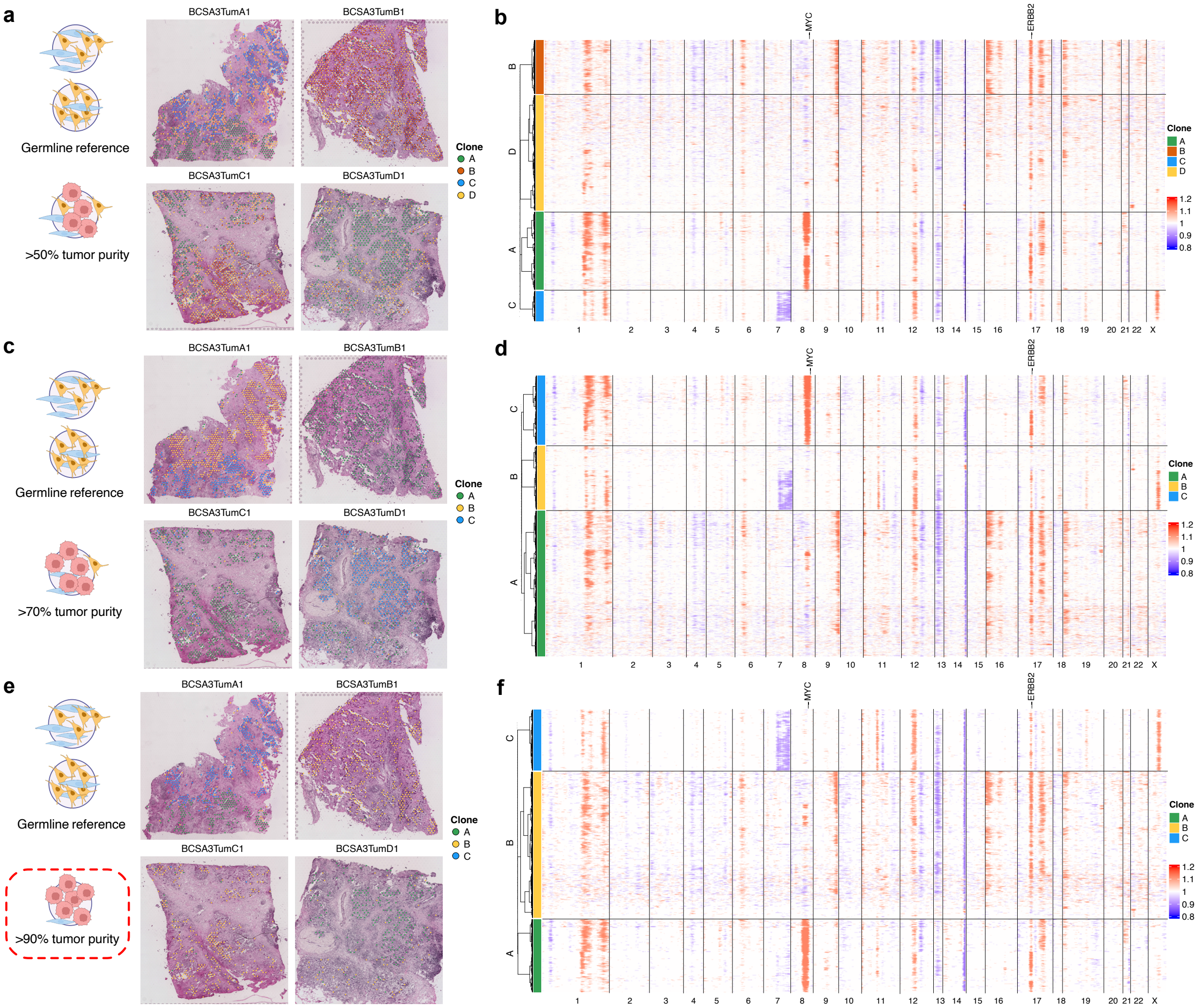

Supplementary Figure 13. Spatial determination of CNV status to identify clonal events in HER2-positive tumor (BCSA3). Spatial visualization of tumor clones on the BCSA3 TumA1, TumB1, TumC1, and TumD1 sections using spots with at least 50% (a), 70% (c), and 90% (e) of tumor purity defined by CTA. Genome-wide CNV analysis was performed for each spot with at least 50% (b), 70% (d), and 90% (f) of tumor cell fractions. The color indicates scaled expression levels, with blue representing copy number loss and red indicating copy number gain. The clonal grouping of spots was defined by hierarchical clustering. The chromosome numbers are displayed at the bottom of the heatmap.

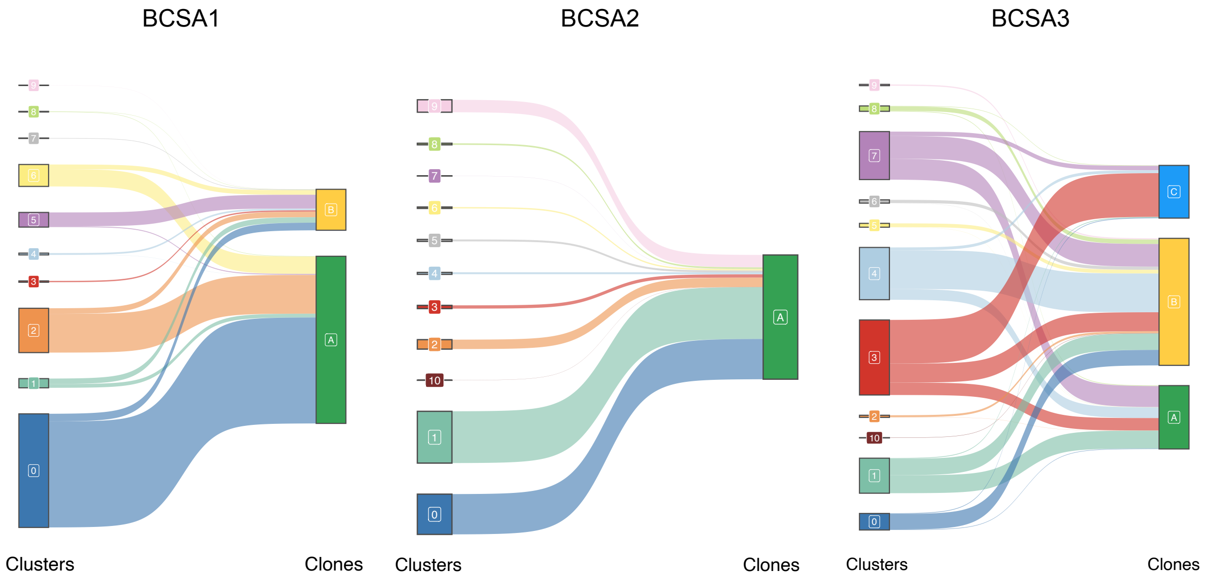

Supplementary Figure 14. Sankey plots showing the distribution of gene expression clusters by inferred clones. The color codes display the gene expression clusters, and the shaded area shows the proportion of spots belonging to the specific clones inferred under the cutoff value of 90% of tumor purity in three tumors.

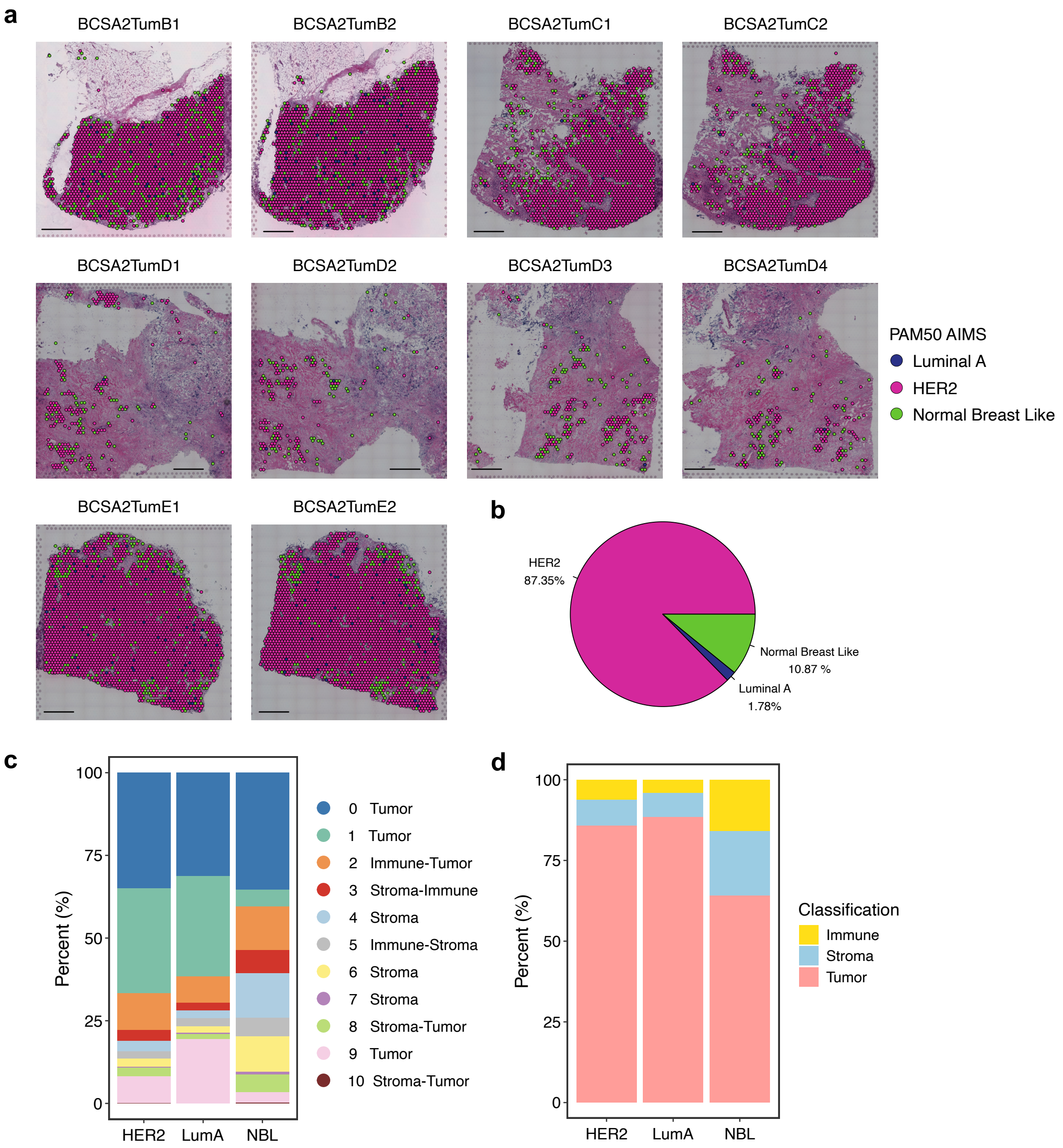

Supplementary Figure 15. Heterogeneity of spatially resolved breast cancer intrinsic subtypes in HER2-positive tumor (BCSA2). (a) The spatially resolved intrinsic subtypes predicted by the AIMS method across all 10 sections of BCSA2. The dark blue color code indicates Luminal A (LumA), fuchsia pink refers to HER2, and green denotes Normal Breast-Like (NBL). Scale bar 1 mm. (b) Pie chart showing the overall distribution of predicted intrinsic subtypes from all BCSA2 sections. (c) Stacked columns display the GEX cluster composition within the HER2, LumA, and NBL subtypes. (d) Percentage of immune, stroma, and tumor cells in each intrinsic subtype predicted by CTA. Immune in yellow, stroma in blue, and tumor in pink.

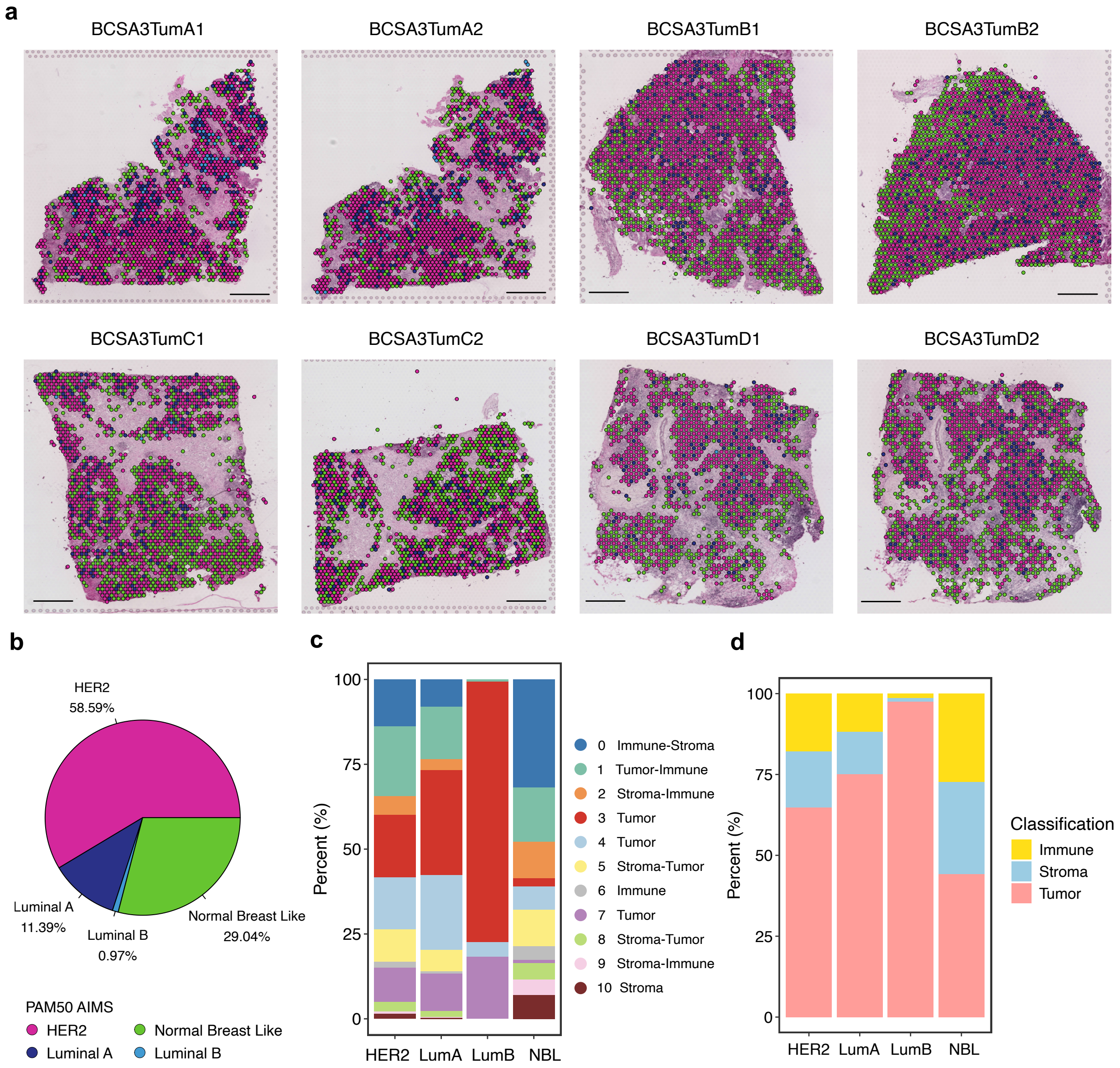

Supplementary Figure 16. Heterogeneity of spatially resolved breast cancer intrinsic subtypes in HER2-positive tumor (BCSA3). (a) The spatially resolved intrinsic subtypes predicted by the AIMS method across all eight sections of BCSA3. The dark blue color code indicates Luminal A (LumA), sky blue represents Luminal B (LumB), fuchsia pink refers to HER2, and green denotes Normal Breast-Like (NBL). Scale bar 1 mm. (b) Pie chart showing the overall distribution of predicted intrinsic subtypes from all BCSA3 sections. (c) Stacked columns displaying the composition of GEX clusters within the HER2, LumA, LumB, and NBL subtypes. (d) Percentage of immune, stroma, and tumor cells in each intrinsic subtype predicted by CTA. Immune in yellow, stroma in blue, and tumor in pink.

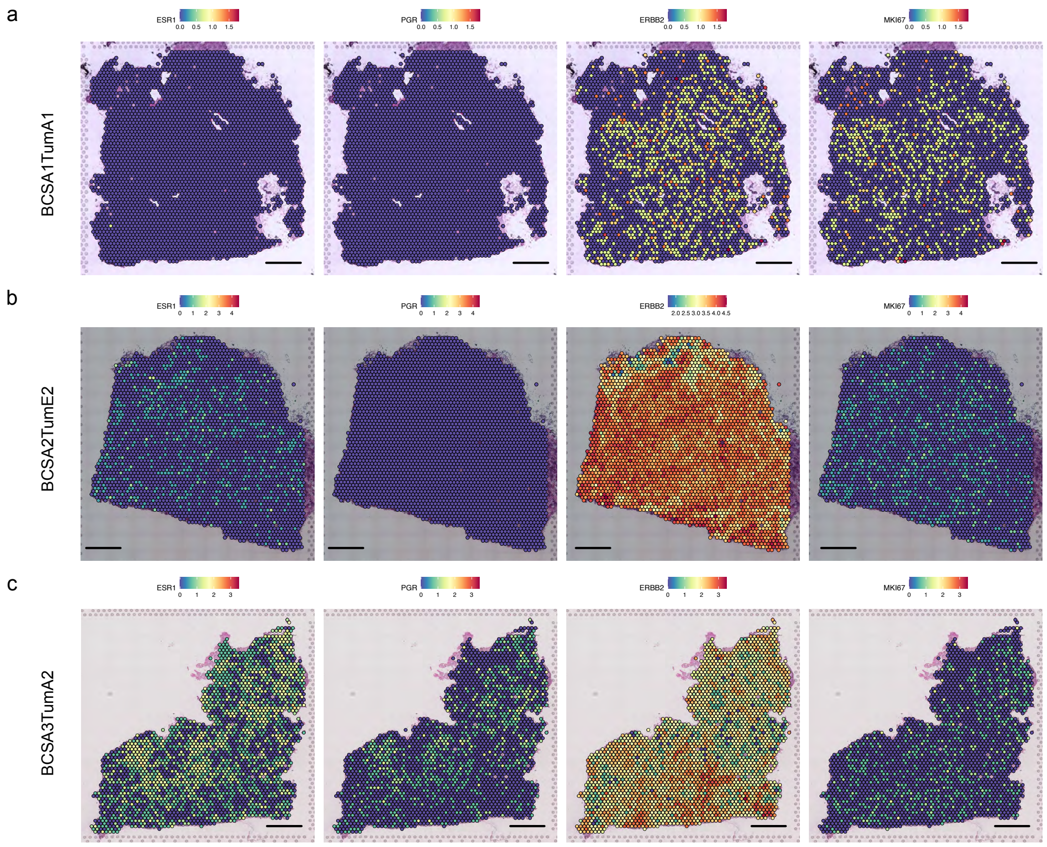

Supplementary Figure 17. Spatial localization and expression of breast cancer-specific biomarkers. Expressions of estrogen receptor alpha (*ESR1*), progesterone receptor (*PGR*), Erb-B2 receptor tyrosine kinase 2 (*ERBB2*), and proliferation marker (*MKi67*) on BCSA1TumA1 (a), BCSA2TumE2 (b), and BCSA3TumA2 (c) samples. Scale bar 1 mm.
